# NR4A family members regulate T cell tolerance to preserve immune homeostasis and suppress autoimmunity

**DOI:** 10.1172/jci.insight.151005

**Published:** 2021-09-08

**Authors:** Ryosuke Hiwa, Hailyn V. Nielsen, James L. Mueller, Ravi Mandla, Julie Zikherman

**Affiliations:** 1 Rosalind Russell and Ephraim P. Engleman Arthritis Research Center, Division of Rheumatology, and; 2 Cardiology Division, Department of Medicine, University of California, San Francisco, San Francisco, California, USA.

**Keywords:** Autoimmunity, Immunology, Autoimmune diseases, T cell development, Tolerance

## Abstract

The NR4A family of orphan nuclear receptors (*Nr4a1*–*3*) plays redundant roles to establish and maintain Treg identity; deletion of multiple family members in the thymus results in Treg deficiency and a severe inflammatory disease. Consequently, it has been challenging to unmask redundant functions of the NR4A family in other immune cells. Here we use a competitive bone marrow chimera strategy, coupled with conditional genetic tools, to rescue Treg homeostasis and unmask such functions. Unexpectedly, chimeras harboring *Nr4a1^–/–^ Nr4a3^–/–^* (double-knockout, DKO) bone marrow developed autoantibodies and a systemic inflammatory disease despite a replete Treg compartment of largely WT origin. This disease differs qualitatively from that seen with Treg deficiency and is B cell extrinsic. Negative selection of DKO thymocytes is profoundly impaired in a cell-intrinsic manner. Consistent with escape of self-reactive T cells into the periphery, DKO T cells with functional, phenotypic, and transcriptional features of anergy accumulated in chimeric mice. Nevertheless, we observed upregulation of genes encoding inflammatory mediators in anergic DKO T cells, and DKO T cells exhibited enhanced capacity for IL-2 production. These studies reveal cell-intrinsic roles for the NR4A family in both central and peripheral T cell tolerance and demonstrate that each is essential to preserve immune homeostasis.

## Graphical abstract

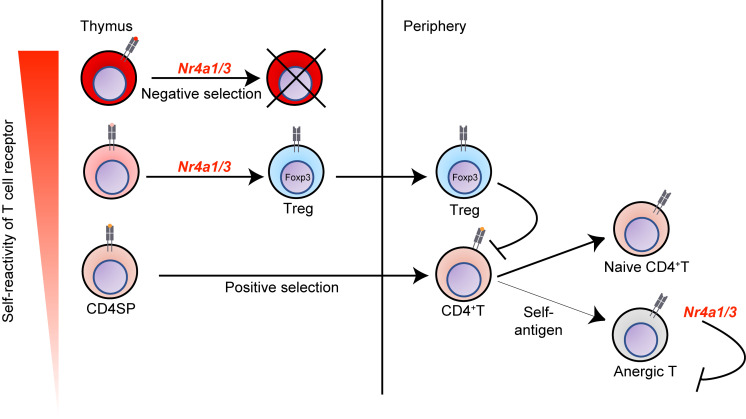

## Introduction

Since the initial discovery of regulatory T cells (Tregs) and their recognition as a distinct T cell lineage dependent upon the transcription factor FOXP3, it has been shown that they are essential for immune homeostasis and tolerance to self (1). Indeed, *Foxp3*-deficient mice and mice with a loss-of-function mutation in *Foxp3* (Scurfy) rapidly develop an autoimmune disease characterized by cytokine storm, immune cell proliferation and infiltration, autoantibody production, and death typically by 4 weeks of age (1–4). Conversely, reintroducing Tregs is sufficient to prevent this disease (2). However, extensive *cell-intrinsic* mechanisms that operate in other immune cell lineages are also essential to maintain tolerance to self, including processes such as deletion and hyporesponsiveness of self-reactive lymphocytes (termed anergy; ref. 5).

Prior work has implicated a small family of orphan nuclear hormone receptors (encoded by *Nr4a1*–*3*) in several of these processes. Most notably, NR4A family members play redundant roles upstream of *Foxp3* to maintain Treg identity and function; deletion of multiple family members in the thymus results in profound Treg deficiency and a severe “Scurfy-like” disease that phenocopies *Foxp3*-deficient mice (6, 7). Therefore, it has been difficult to isolate redundant functions of this family in other immune cell populations. Yet this remains an important area to explore since the NR4A family are widely expressed and thought to be druggable targets that may facilitate manipulation of immune cell function in autoimmune disease, tumor immunotherapy, and hematologic malignancies (8–11).

*Nr4a1*–*3* (encoding NUR77, NURR1, and NOR-1, respectively) are rapidly upregulated in response to mitogenic stimuli, including antigen receptor ligation, and thought to function as constitutively active transcription factors without a confirmed endogenous ligand (12). As a result, these family members are upregulated in T and B cells after acute antigen encounter, in Tregs in the steady state, in thymocytes undergoing negative selection, and in self-reactive, anergic, or exhausted lymphocytes in response to chronic antigen stimulation (6, 9, 13–19). Indeed, the NR4A family has been argued to play a tolerogenic role in all these contexts. The NR4A family selectively restrains the survival and expansion of B cells that encounter antigen (signal 1) in the absence of costimulation (signal 2; refs. 15, 20). Similarly, overexpression of *Nr4a1* or *Nr4a3* mediates antigen-induced cell death in the thymus, while a dominant-negative transgenic (Tg) construct has the opposite effect (13, 21, 22). However, *Nr4a1^–/–^* mice exhibit extremely subtle defects in thymic negative selection (23, 24), suggesting possible redundancy among the family members. *Nr4a1* and *Nr4a3* also play nonredundant roles in peripheral conventional T cells (Tconv): most notable among these are roles for *Nr4a1* in CD4^+^ T cell anergy (17) and an additive role for all 3 family members in CD8^+^ T cell exhaustion (9). Finally, it has been argued that *Nr4a1* and *Nr4a3* redundantly maintain myeloid homeostasis since, in their absence, a myeloproliferative disease is observed (25). However, unmasking redundancy between NR4A family members in many of these contexts has been hampered by profound immune dysregulation that develops in the absence of functional Tregs.

We sought to bypass this obstacle by generating competitive bone marrow (BM) chimeras harboring both wild-type (WT) cells (that could reconstitute a functional Treg compartment) and double-knockout (DKO) cells (lacking both *Nr4a1* and *Nr4a3*) to isolate cell-intrinsic immune functions for the NR4A family. Unexpectedly, mixed chimeras harboring both WT and DKO BM rapidly developed antinuclear autoantibodies (ANAs) and a systemic inflammatory disease, despite a replete Treg compartment of largely WT origin. The disease that developed in BM chimeras was B cell extrinsic and qualitatively different from that in germline DKO mice. We found that negative selection of DKO thymocytes in competitive chimeras was profoundly impaired in a cell-autonomous manner. DKO Tconv cells with phenotypic, functional, and transcriptional features of antigen experience and anergy accumulate in these chimeras, suggesting escape of self-reactive T cells into the periphery. However, self-reactive DKO CD4^+^ Tconv cells nevertheless exhibited expression of inflammatory mediators and exaggerated capacity for IL-2 production, suggesting that anergy was defective. Our findings unmask essential, redundant roles for the NR4A family in central and peripheral T cell tolerance to maintain immune homeostasis.

## Results

### Systemic immune dysregulation in mice with germline deficiency of Nr4a1 and Nr4a3.

*Nr4a1*, -*2*, and -*3* are expressed in thymocytes, Tregs, and peripheral T cells, but *Nr4a2*’s expression is minimal under steady-state conditions (Supplemental Figure 1A; supplemental material available online with this article; https://doi.org/10.1172/jci.insight.151005DS1; created using data from ImmGen database, https://www.immgen.org). To unmask redundant functions of the NR4A family, we generated mice lacking germline expression of both *Nr4a1* and *Nr4a3*. We used *Nr4a1^fl/fl^* mice to generate *Nr4a1*-deficient mice with germline excision of the *loxp*-flanked locus and bred this with a CRISPR-generated *Nr4a3^–/–^* line that we recently described (20). An independently generated line of *Nr4a1^–/–^* mice in widespread use has been reported to express a truncated NUR77 protein encoded by exon 2 of *Nr4a1* (23, 26). Our generated *Nr4a1^–/–^ Nr4a3^–/–^* mice (germline DKO, denoted as gDKO herein) do not express exon 2 of *Nr4a1* consistent with the prior analysis of *Nr4a1^fl/fl^* mice (26).

**Figure 1:**
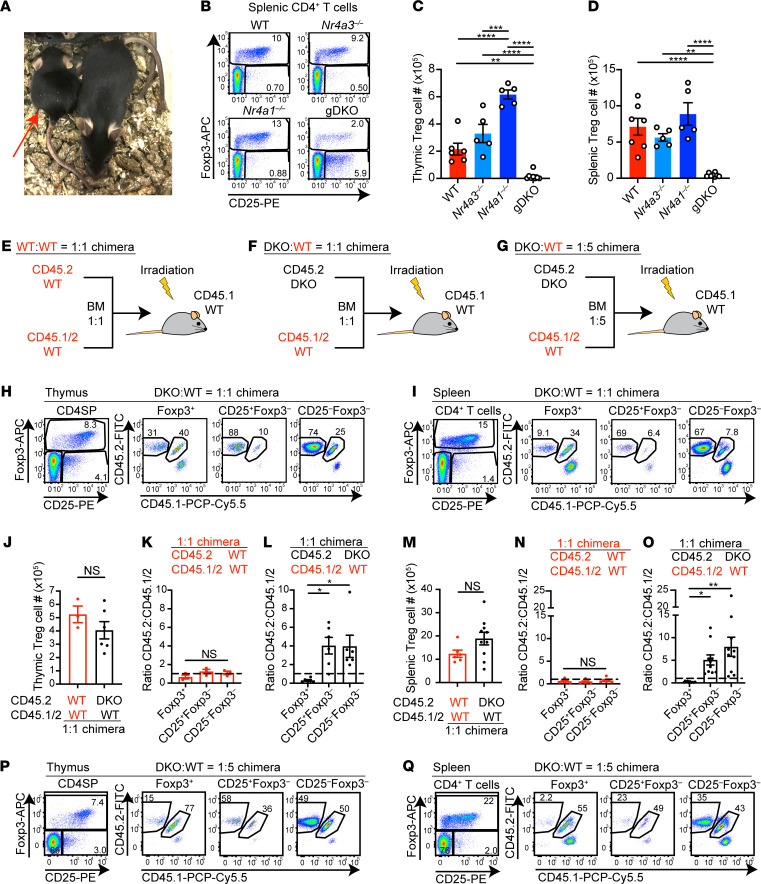
Systemic immune dysregulation and Treg deficiency in mice with germline deficiency of *Nr4a1* and *Nr4a3*. (**A**) *Nr4a1^–/–^Nr4a3^–/–^* (gDKO) mouse (red arrow) compared with healthy littermate, 4 weeks; representative of *n* = 8. (**B**) Flow plots show splenic CD4^+^ T cells with FOXP3^+^ Treg gate in mice of each genotype. Representative of 5 mice/genotype. (**C** and **D**) Quantification of thymic (**C**) and splenic (**D**) Treg cell number (*n* = 5, 3- to 4-week-old gDKO and 5- to 6-week-old mice with other genotypes). (**E**–**G**) Competitive BM chimera design. (**H** and **I**) Flow plots show thymic CD4 single-positive (CD4SP) (**H**) or splenic CD4^+^ T cell (**I**) subpopulations in 1:1 DKO:WT chimeras. Representative of 6 (**H**) or 10 (**I**) chimeras. (**J**–**O**) Quantification of thymic (**J**) or splenic (**M**) Treg cell number in 1:1 chimeras. Ratio of CD45.2 to CD45.1/2 for thymic (**K** and **L**) or splenic (**N** and **O**) Treg, CD25^+^FOXP3^–^, and CD25^–^FOXP3^–^ cells in 1:1 chimeras, normalized to double-positive (DP) thymocytes. *n* = 3 (**J**–**L**) or 6 (**M**–**O**), pooled from 2 sets of independently generated chimeras 6–10 weeks posttransplant. (**P** and **Q**) Flow plots show thymic CD4SP (**P**) or splenic CD4^+^ T cell (**Q**) subpopulations in 1:5 DKO:WT chimera. Representative of ≥3 chimeras from 1 chimera setup. Graphs depict mean ± SEM. Statistical significance was assessed by 1-way ANOVA with Tukey’s test (**C**, **D**, **K**, **L**, **N**, and **O**) or 2-tailed unpaired Student’s *t* test (**J** and **M**). *P < 0.05; **P < 0.01; ***P < 0.001; ****P < 0.0001. NS, not significant.

gDKO mice were born at Mendelian ratios but exhibited severe runting (Figure 1A) and invariably died before 4 weeks of age, consistent with observed mortality in an independent gDKO line generated with distinct *Nr4a1*- and *Nr4a3*-null alleles (25). As previously reported for CD4-cre *Nr4a^fl/fl^* *Nr4a3^–/–^* mice (6), our gDKO mice exhibited near-complete loss of FOXP3^+^ Tregs in both thymus and periphery (Figure 1, B–D). gDKO mice also exhibited severe thymic atrophy, but loss of peripheral Tregs was disproportionate relative to a more modest reduction of total splenocytes (Supplemental Figure 1, B and C). Concurrently, we observed expansion of a unique population of CD4^+^CD25^+^FOXP3^–^ T cells in the thymus and the periphery that may represent cells that have either lost or failed to upregulate expression of FOXP3, as described elsewhere (Figure 1B; Supplemental Figure 1, D–G; and refs. 6, 27, 28). Importantly, and consistent with prior reports, neither *Nr4a1^–/–^* nor *Nr4a3^–/–^* single-knockout (SKO) mice exhibited Treg loss, expansion of this unique cell population, or frank disease (Figure 1, B–D; Supplemental Figure 1, B–G; and ref. 6).

### Cell-intrinsic Treg defect in the absence of Nr4a1 and Nr4a3.

Systemic inflammatory disease and associated thymic atrophy preclude the study of thymic development and mature Tconv cells in gDKO mice (Supplemental Figure 1B). Similar mortality observed in both germline (25) and CD4-cre conditional mouse lines (6) suggested that disease in gDKO animals might be due to Treg deficiency in both settings. We reasoned that restoring functional Tregs could unmask cell-intrinsic functions of NR4A family in other immune cell populations. To do so, we generated competitive chimeras to allow WT donor BM to reconstitute a functional Treg compartment. Equal proportions of congenically marked donor BM from CD45.2 gDKO and CD45.1/2 WT mice were transplanted into lethally irradiated CD45.1 BoyJ recipients (Figure 1F). In parallel, we generated control chimeras in which CD45.1 hosts were reconstituted with a mixture of CD45.2 WT and CD45.1/2 WT BM (Figure 1E). In addition, we generated mixed chimeras with a low proportion of gDKO donor BM (1:5 ratio) to further ensure development of a WT Treg compartment (Figure 1G). We assessed reconstitution and immune phenotypes of chimeras at sequential points between 6 and 14 weeks posttransplant.

Consistent with studies of CD4-cre chimeras, we observed a profound cell-intrinsic disadvantage for DKO Tregs in the thymus and spleen when compared with CD4SP thymocytes (Figure 1, H–O, and ref. 6). Similar results were attained with DKO:WT 1:5 chimera (Figure 1, P and Q; and Supplemental Figure 1, H–K). FOXP3 and CD25 expression in DKO Tregs was reduced (Supplemental Figure 1, L–O), consistent with a role for the NR4A family in “maintenance” of Treg identity (18). Most importantly, the Treg compartment was restored, and Treg number was comparable between DKO:WT chimera and WT:WT chimera (Figure 1, J and M; and Supplemental Figure 1, J and K). We also confirmed Tregs were largely reconstituted from WT donors in DKO:WT 1:5 chimeras (Figure 1, P and Q). This allowed us to explore the cell-intrinsic roles of the NR4A family in other immune cell types.

### Thymic atrophy is partially rescued in competitive chimeras.

**Figure 2:**
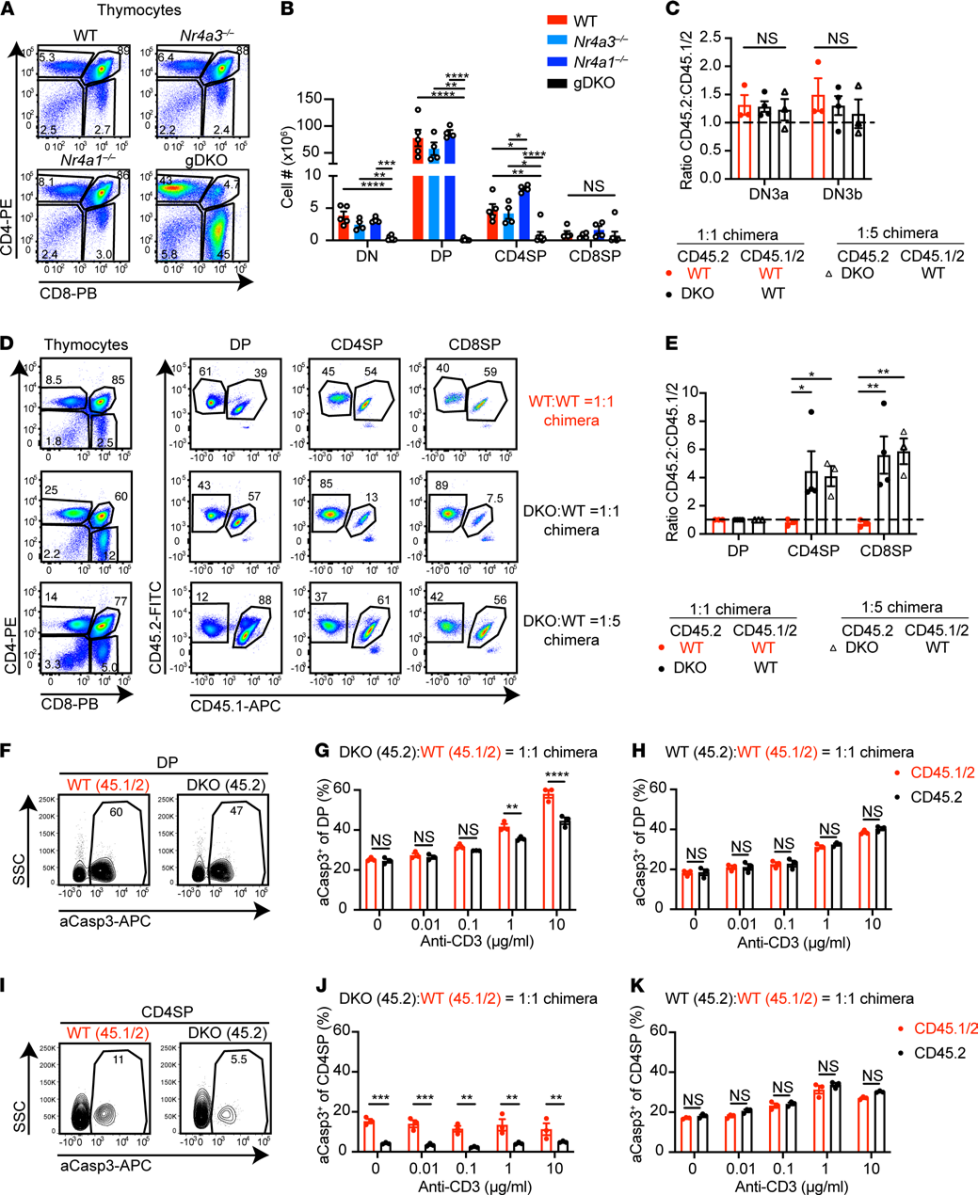
DKO thymocytes have a cell-intrinsic defect in negative selection. (**A**) Flow plots show thymic subsets in WT, *Nr4a3^–/–^*, *Nr4a1^–/–^*, and gDKO mice. Representative of *n* ≥ 4 mice/genotype. (**B**) Quantification of thymic subset cell number as gated in **A**; (*n* ≥ 4, 3 to 4-week-old gDKO and 5- to 6-week-old mice of other genotypes). (**C**) Ratio of CD45.2 to CD45.1/2 thymocytes among thymic DN3a and DN3b subsets (as gated in Supplemental Figure 2B), normalized to DN2 subset (*n* = 3–4 chimeras). (**D**) Flow plots show thymic subsets in competitive chimeras. Representative of ≥3 mice/genotype. (**E**) Ratio of CD45.2 to CD45.1/2 thymic subsets as gated in **D** normalized to DP subset (*n* ≥ 3). Data in **C**–**E** were from 6 to 7 weeks posttransplant chimeras pooled from 3 sets of independently generated chimeras. (**F**–**K**) Thymocytes from 1:1 DKO:WT chimeras were cultured with varying doses of plate-bound anti-CD3 and 2 μg/mL of anti-CD28 for 24 hours. Cells were stained to detect CD4/CD8 surface markers, followed by permeabilization and detection of active Caspase3 (aCasp3). Representative plots show aCasp3 expression in WT CD45.1/2 and DKO CD45.2 DP (**F**) and CD4SP (**I**) thymocytes from 1:1 DKO chimeras cultured with 10 μg/mL anti-CD3. Quantification percentage aCasp3^+^ cells among DP (**G** and **H**) or CD4SP (**J** and **K**) in 1:1 DKO:WT (**G** and **J**) or 1:1 WT:WT (**H** and **K**) chimeras (*n* = 3 from 1 chimera setup). Graphs depict mean ± SEM. Statistical significance was assessed by 1-way (**B**) or 2-way (**C** and **E**) ANOVA with Tukey’s test or 2-tailed unpaired Student’s *t* test with the Holm-Šídák method (**G**, **H**, **J**, and **K**). *P < 0.05; **P < 0.01; ***P < 0.001; ****P < 0.0001. NS, not significant.

gDKO mice exhibited severe thymic atrophy with marked reduction of all thymocyte subsets and disproportionate loss of double-positive (DP) thymocytes (Figure 2, A and B). We postulated that this might be an indirect consequence of Treg deficiency and systemic inflammation in gDKO mice, since DP thymocytes are sensitive to glucocorticoid-induced apoptosis (29). Indeed, profound thymic atrophy was partially rescued in DKO:WT 1:1 chimeras and fully rescued in DKO:WT 1:5 chimeras within the first 6 weeks of reconstitution (Supplemental Figure 2A). However, progressive thymic atrophy was observed over time in DKO:WT chimeras (relative to WT:WT control chimera). This led us to focus on early time points to isolate cell-intrinsic roles for the NR4A family during thymic development (6 weeks posttransplant).

### NR4A expression is dispensable for thymic β-selection.

We previously showed, using a fluorescent reporter of *Nr4a1* transcription (NUR77-eGFP), that GFP is upregulated at the β-selection checkpoint during thymic development, suggesting *Nr4a1* and family members might play a functional role here (14). Immature double-negative (DN) thymocytes (lacking both CD4 and CD8 expression) recombine the TCRβ chain, which pairs with pre-TCRα to signal in an antigen-independent manner at the “β-selection” checkpoint (30). This occurs during the DN3 stage of development; preselection DN3a thymocytes are CD25^hi^CD44^lo^ and FSC^lo^, while DN3b thymocytes that have traversed this checkpoint successfully express the same surface markers but are larger (FSC^hi^; ref. 31 and Supplemental Figure 2B). We probed β-selection in both 1:1 and 1:5 DKO:WT competitive chimeras but identified no advantage for either DKO or WT CD45.2 cells relative to competitor CD45.1/2 WT cells (Figure 2C).

### DKO thymocytes have a profound cell-intrinsic defect in negative selection.

Studies of 2 independent NUR77-eGFP reporter lines as well as transcriptional analysis have shown that *Nr4a* genes are upregulated at the positive selection checkpoint and are especially enriched among thymocytes undergoing negative selection (14, 18, 32). Overexpression of full-length and truncated dominant-negative constructs suggested that NUR77 and NOR-1 redundantly mediate thymic negative selection (13, 22, 33, 34), yet *Nr4a1^–/–^* mice exhibit only subtle defects (23, 24). We reasoned that DKO:WT competitive chimeras could unmask cell-intrinsic, redundant functions of the NR4A family during thymic selection. Indeed, we observed a striking advantage for DKO cells in CD4SP and CD8SP subsets relative to DP in 1:1 and 1:5 chimeras but not at an earlier stage (Figure 2, D and E), suggesting enhanced positive selection or impaired negative selection. However, we saw no advantage for DKO cells in postselection DP thymocytes relative to preselection DP thymocytes, arguing against a role during positive selection (Supplemental Figure 2, C and D).

To test the hypothesis that DKO thymocytes escape negative selection, we assessed antigen-induced apoptosis by detection of activated Caspase 3 (aCasp3) in thymocytes from chimeras. We observed reduced aCasp3^+^ DKO relative to WT thymocytes after in vitro TCR stimulation (Figure 2, F, G, I, and J). By contrast, we saw no difference between donors in control WT:WT chimeras (Figure 2, H and K). Notably, we also saw no significant difference in aCasp3 expression in SKO thymocytes relative to cocultured WT (Supplemental Figure 2, E and F). Moreover, mixed chimeras generated with *Nr4a1^–/–^* or *Nr4a3^–/–^* SKO mice revealed only a small competitive advantage for CD8SP cells, suggesting a largely redundant role for these family members during negative selection that is only unmasked when both family members are lost (ref. 35 and Supplemental Figure 2G). We conclude that DKO thymocytes escape negative selection and show for the first time to our knowledge that this is a profound effect in a physiological context, independent of either a TCR-Tg or NR4A misexpression.

### Myeloproliferative disorder in DKO mice is a non–cell-autonomous effect of NR4A deficiency.

**Figure 3:**
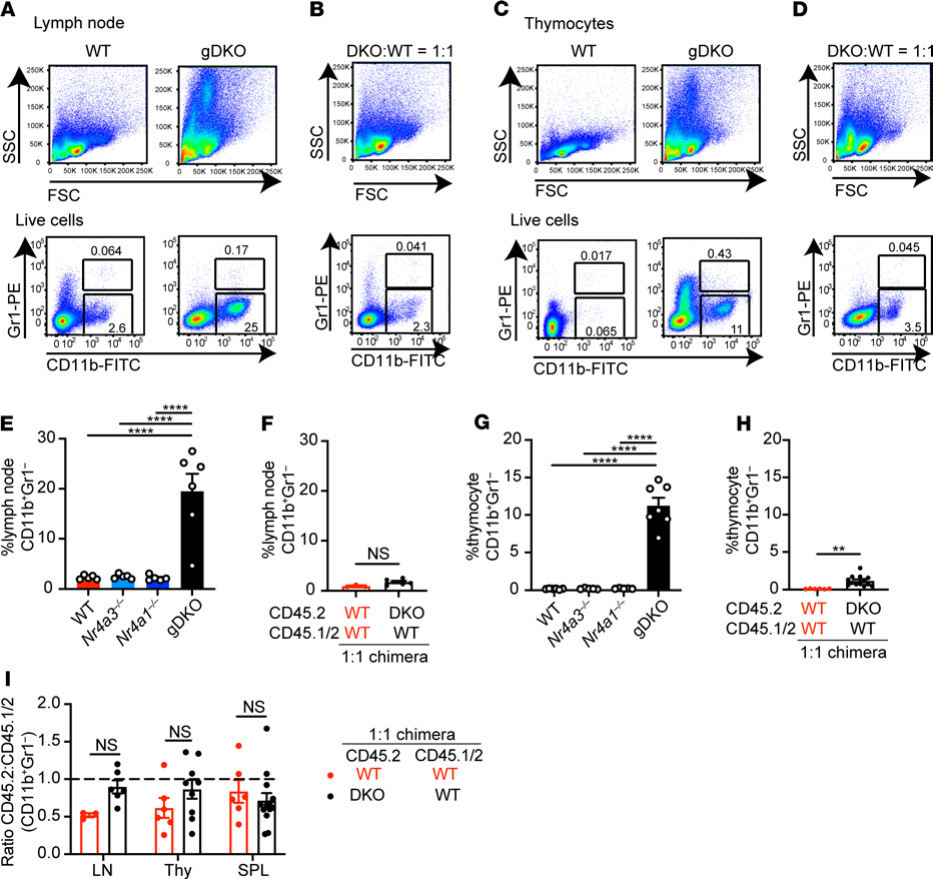
Myeloproliferative disorder in DKO mice is a non–cell-autonomous effect of NR4A deficiency. (**A**–**D**) Lymph node cells (**A** and **B**) and thymocytes (**C** and **D**) from WT and gDKO mice (**A** and **C**) or 1:1 DKO:WT chimeras (**B** and **D**) were stained to detect CD11b and Gr1 (Ly6G/Ly6C) expression. Shown are representative plots of ≥ 5 mice. (**E**–**H**) Quantification of CD11b^+^Gr1^–^ cells in lymph nodes (**E** and **F**) and thymocytes (**G** and **H**) from WT, *Nr4a3^–/–^*, *Nr4a1^–/–^*, and gDKO mice (**E** and **G**) (*n* ≥ 5, 3- to 4-week-old gDKO and 5- to 6-week-old mice of other genotypes) and from WT:WT = 1:1 and DKO:WT = 1:1 chimeras (**F** and **H**) (*n* ≥ 3 pooled from 2 sets of independently generated chimeras). (**I**) Ratio of CD45.2 to CD45.1/2 for CD11b^+^Gr1^–^ cells in lymph nodes, thymus, and spleen from WT:WT = 1:1 and DKO:WT = 1:1 chimera (*n* ≥ 3 pooled from 2 sets of independently generated chimeras). Graphs depict mean ± SEM. Statistical significance was assessed by 1-way ANOVA with Tukey’s test (**E** and **G**), 2-tailed unpaired Student’s *t* test with (**I**) or without (**F** and **H**) the Holm-Šídák method. **P < 0.01; ****P < 0.0001. NS, not significant.

Previous studies report that a severe myeloproliferative disorder develops in the first weeks of life in independently generated gDKO mice (25). This was not seen in SKO animals lacking 1 *Nr4a* family member, although mice lacking 3 out of 4 *Nr4a* alleles (i.e., *Nr4a1^+/–^ Nr4a3^–/–^* or *Nr4a1^–/–^ Nr4a3^+/–^*) did eventually succumb to a similar disease at much later points (36). Consistent with this, we observed profound expansion of SSC^hi^ cells infiltrating all hematopoietic tissues and lymphoid organs in gDKO mice; this not only included BM and spleen (Supplemental Figure 3) but also was especially pronounced in lymph nodes and thymus (Figure 3, A–D). These SSC^hi^ cells are CD11b^+^ but largely Gr1^–^. Since Treg-deficient animal models like Scurfy and *Foxp3*-deficient mice similarly exhibit myeloid expansion (2, 3, 37), we hypothesized that the myeloproliferative disorder observed in gDKO animals was due to Treg deficiency. Consistent with this possibility, myeloid expansion is observed in CD4-cre *Nr4a1^fl/fl^ Nr4a3^–/–^* mice but not in mixed chimeras generated with WT donor BM (6). Resolving this question with gDKO cells has important implications since the NR4A family may represent important drug targets in myeloid leukemic diseases (8, 25, 36). Indeed, in our DKO:WT chimeras, myeloid expansion was suppressed (even after 12 weeks of reconstitution), and DKO cells exhibited no competitive advantage in these compartments (Figure 3, A–I; and Supplemental Figure 3, A, B, D, and E). We did observe a minor infiltration of SSC^hi^CD11b^+^ cells into the thymus of 1:1 DKO:WT but not WT:WT chimeras, and here as well the effect of *Nr4a* deficiency was cell extrinsic (Figure 3, D, H, and I). Taken together, these data support our hypothesis that the myeloproliferative disorder observed in gDKO animals is due to a non–cell-autonomous impact of *Nr4a* deletion.

### Abnormal B cell homeostasis in DKO mice is a non–cell-autonomous effect of NR4A deficiency.

**Figure 4:**
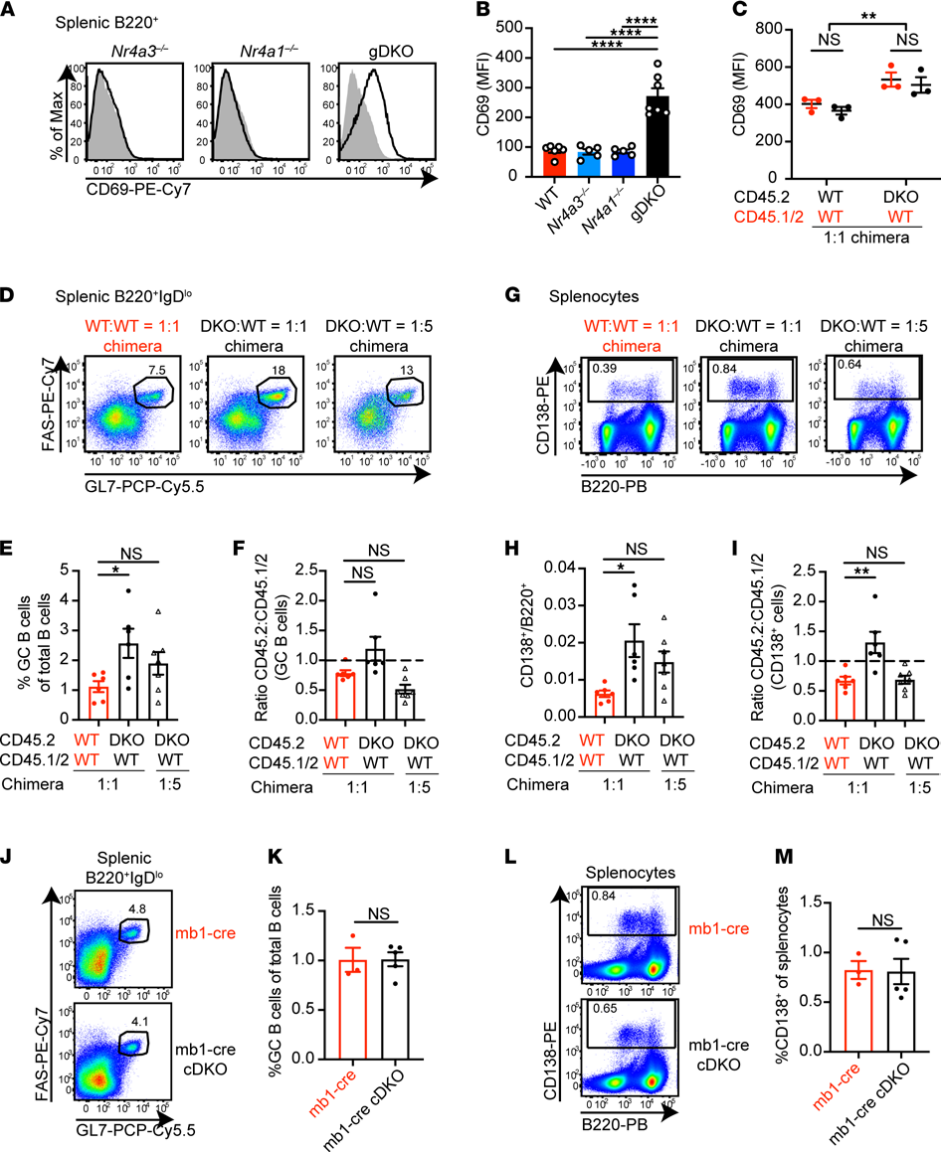
Abnormal B cell homeostasis in DKO mice is a non–cell-autonomous effect of NR4A deficiency. (**A**) Representative flow plots showing CD69 expression on splenic B cells from WT (shaded gray histogram) and overlaid *Nr4a3^–/–^*, *Nr4a1^–/–^*, or gDKO mice. (**B**) Quantification of CD69 MFI as in **A** (data in **A** and **B** represent *n* ≥ 5, 3- to 4-week-old gDKO and 5- to 6-week-old mice of other genotypes). (**C**) Quantification of CD69 MFI on splenic B cells of each donor genotype in competitive 1:1 chimeras (*n* = 3 from 1 chimera setup). (**D** and **G**) Representative flow plots show FAS^hi^GL7^+^ GC B cells pregated on B220^+^IgD^lo^ splenocytes (**D**) and CD138^+^ splenocytes (**G**) from competitive chimeras. (**E**–**I**) Frequency of GC B cells among total B cells (**E**), ratio of CD45.2 to CD45.1/2 GC B cells normalized to B220^+^IgD^hi^ naive B cells (**F**), ratio of CD138^+^ to B220^+^ splenocytes (**H**), ratio of CD45.2 to CD45.1/2 CD138^+^ cells normalized to B220^+^CD138^–^ cells (**I**) from competitive chimeras as gated in **D** and **G** (data in **D**–**I** represent *n* ≥ 6 pooled from 3 sets of independently generated chimeras). (**J**–**M**) Representative flow plots show GC B cells (**J**) and CD138^+^ cells (**L**) in spleen from host chimeras transplanted with either mb1-cre or mb1-cre *Nr4a1^fl/fl^ Nr4a3^–/–^* (cDKO) BM after 40 weeks. Frequency of GC B cells among total B cells (**K**) and CD138^+^ cells among splenocytes (**M**) (*n* ≥ 3). Graphs depict mean ± SEM. Statistical significance was assessed by 1-way ANOVA with Tukey’s test (**B**) or Dunnett’s test (**E**, **F**, **H**, and **I**) or 2-tailed unpaired Student’s *t* test with (**C**) or without (**K** and **M**) the Holm-Šídák method. *P < 0.05; **P < 0.01; ****P < 0.0001. NS, not significant.

Like other Treg-deficient models, gDKO mice exhibit spontaneous polyclonal B cell activation and differentiation under steady-state conditions (Figure 4, A and B; and Supplemental Figure 4, A–F). We recently identified a cell-intrinsic role for the NR4A family in restraining antigen-induced B cell expansion in the absence of costimulation, including in the context of B cell tolerance (15, 20). We therefore sought to determine to what extent spontaneous B cell activation and differentiation in gDKO mice (under homeostatic conditions) were attributable to a B cell–intrinsic role for the NR4A family.

Chimeras did not reveal a competitive advantage or disadvantage for DKO cells during splenic B cell development apart from a subtle disadvantage in the marginal zone compartment (Supplemental Figure 4G and ref. 20). B cells in 1:1 DKO:WT chimeras expressed higher levels of activation markers than B cells in WT:WT chimeras (Figure 4C and Supplemental Figure 4, H and I). However, this did not differ between donor genotypes within individual chimeras, suggesting a B cell–extrinsic effect of NR4A deficiency. We observed expansion of germinal center (GC) B cells and CD138^+^ cells in DKO:WT chimeras relative to WT:WT control chimeras, but this was similarly B cell extrinsic (Figure 4, D–I). Indeed, no expansion of the GC or CD138^+^ compartment was evident under steady-state conditions in mice lacking *Nr4a1/3* exclusively in the B cell compartment (mb1-cre *Nr4a1^fl/fl^ Nr4a3^–/–^*), even when aged to 40 weeks (Figure 4, J–M). Nor could we detect an advantage for mb1-cre DKO B cells in a competitive setting (Supplemental Figure 4, J–L). We conclude that there is evidence of a spontaneous polyclonal B cell activation and differentiation in gDKO chimeras, but it is a non–B cell–autonomous effect of NR4A deficiency.

### Reconstitution of WT Tregs in competitive chimeras does not rescue DKO CD8^+^ T cell homeostasis.

**Figure 5:**
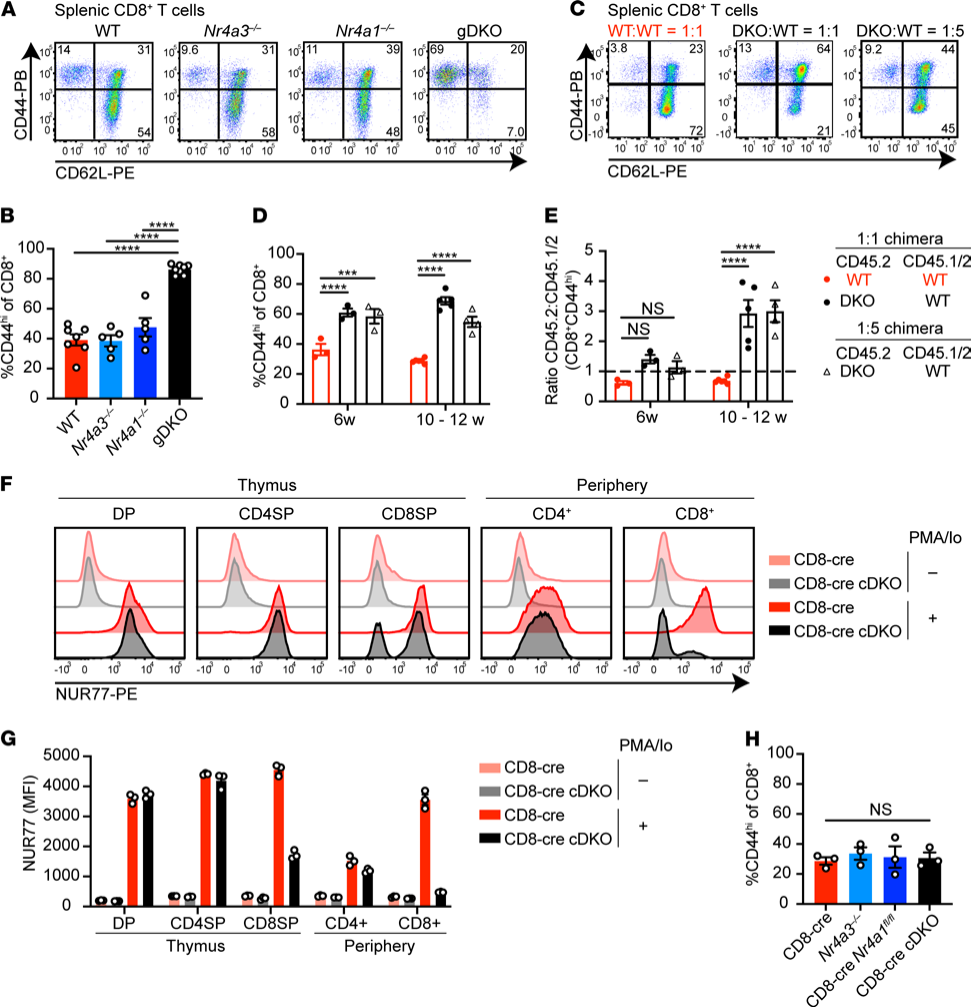
Reconstitution of WT Treg compartment does not restore CD8^+^ T cell homeostasis in competitive chimeras. (**A**) Splenocytes from WT, *Nr4a3^–/–^*, *Nr4a1^–/–^*, and gDKO mice were stained to detect CD8^+^ T cell subsets on the basis of CD44 and CD62L expression. Plots are representative of ≥5 mice/genotype. (**B**) Quantification of splenic CD44^hi^CD8^+^ T cells as gated in **A** (*n* ≥ 5, 3- to 4-week-old gDKO and 5- to 6-week-old mice of other genotypes). (**C**) Flow plots showing the peripheral CD8^+^ T cell subsets in competitive chimeras, as described for **A** above. Representative of ≥7 chimeras of each type. (**D**) Quantification of splenic CD44^hi^CD8^+^ T cells from chimeras as gated in **C** at varied time points posttransplant (*n* ≥ 3). (**E**) Ratio of CD45.2 to CD45.1/2 for CD8^+^CD44^hi^ population as gated in **C**, normalized to naive CD8^+^CD44^lo^CD62L^hi^ gate (*n* ≥ 3). Data in **C**–**E** pooled from 2 sets of independently generated chimeras. (**F** and **G**) Thymocytes and splenocytes from CD8-cre and CD8-cre *Nr4a1^fl/fl^ Nr4a3^–/–^* (cDKO) mice were stimulated with PMA and ionomycin (PMA/Io) for 2 hours. Flow plots show intracellular NUR77 expression following fixation and permeabilization within thymic and splenic T cell subsets (**F**). Quantification of NUR77 MFI in T cell subsets (**G**) (*n* = 3 mice/genotype). (**H**) Quantification of splenic CD8^+^CD44^hi^ T cells from CD8-cre, *Nr4a3^–/–^*, CD8-cre *Nr4a1^fl/fl^*, and CD8-cre cDKO mice (*n* = 3 mice/genotype). Graphs depict mean ± SEM. Statistical significance was assessed by 1-way ANOVA with Tukey’s test (**B** and **H**) or 2-way ANOVA with Dunnett’s test (**D** and **E**). ***P < 0.001; ****P < 0.0001. NS, not significant.

Progressive thymic atrophy (Supplemental Figure 2A) and spontaneous B activation and differentiation in DKO:WT chimeras (Figure 4) suggested the development of a systemic autoimmune and inflammatory state despite replete and largely WT Treg compartment. We next probed the mature T cell compartment to understand the source of this immune dysregulation. gDKO mice exhibited an expanded effector memory compartment and nearly complete loss of naive CD8^+^ T cells (Figure 5, A and B; and Supplemental Figure 5, A and B). However, despite reconstitution of a replete Treg compartment of WT origin (Figure 1M), DKO:WT chimeras nevertheless exhibited marked accumulation of CD44^hi^CD8^+^ T cells relative to WT:WT control chimeras (Figure 5, C and D), and moreover, DKO T cells accumulated in this compartment (Figure 5E). In addition, these CD44^hi^CD8^+^ DKO T cells upregulated programmed cell death 1 (PD-1) expression, suggesting an exhausted state (Supplemental Figure 5, C and D). These observations reveal a T cell–intrinsic role for the NR4A family in CD8^+^ T cell homeostasis.

### Abnormal DKO CD8^+^ T cell homeostasis in competitive chimeras is due to a cell-intrinsic role for Nr4a1 and Nr4a3 during thymic development.

To test whether abnormal DKO CD8^+^ T cell homeostasis reflects a requirement for the NR4A family during thymic selection or exclusively in the periphery, we took advantage of a CD8-cre construct driven by the E8I enhancer that expresses specifically in mature CD8SP and peripheral CD8^+^ T cells to generate CD8-cre *Nr4a1^fl/fl^ Nr4a3^–/–^* mice (CD8-cre cDKO; ref. 38 and Figure 5, F and G). We can confirm that this cre is not active until after thymic DP stage and positive selection checkpoint are traversed because NUR77 expression in the mature CD4 lineage of CD8-cre cDKO mice was intact (Figure 5, F and G). Since accumulation of CD44^hi^CD8^+^ T cells was not observed in CD8-cre cDKO mice (Figure 5H and Supplemental Figure 5E), we conclude that this phenotype must be attributable to a role for the NR4A family earlier in development and likely reflects escape of self-reactive CD8^+^ T cells into the periphery due to impaired negative selection.

### Cell-intrinsic accumulation of CD4^+^ DKO T cells with anergic phenotype in competitive chimeras.

**Figure 6:**
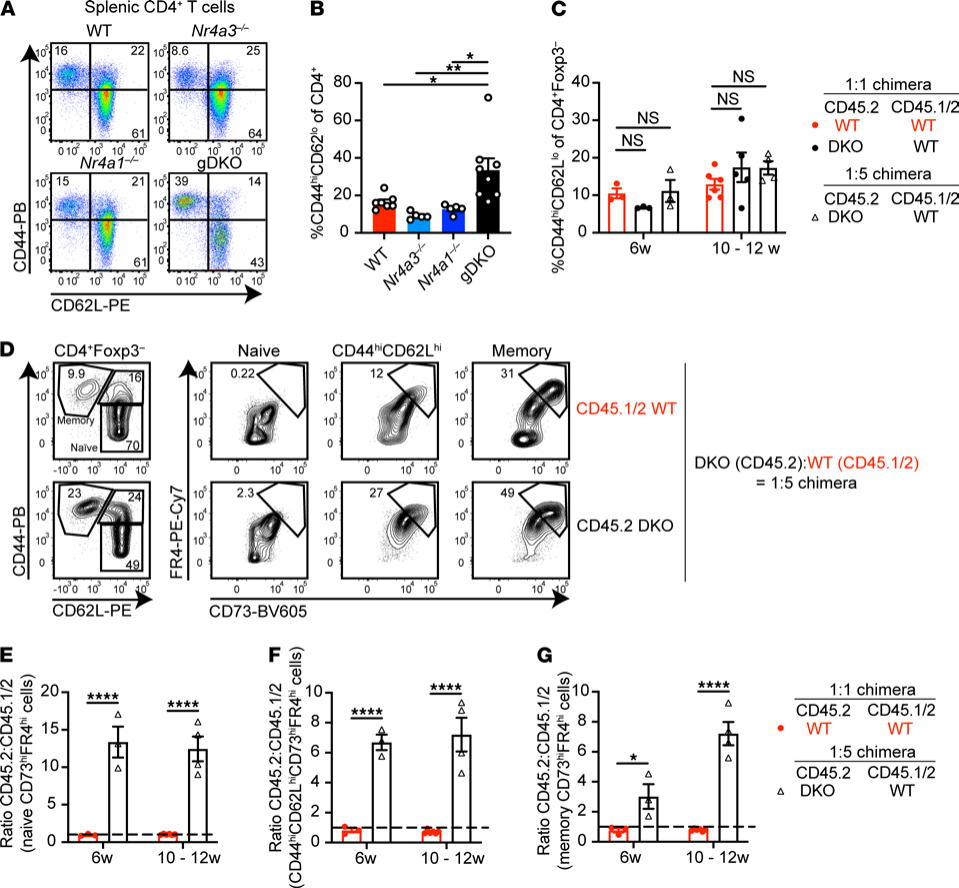
Accumulation of anergic DKO CD4^+^ T cells in competitive chimeras. (**A**) Splenocytes from WT, *Nr4a3^–/–^*, *Nr4a1^–/–^*, and gDKO mice were stained to detect CD4^+^ T cell subsets on the basis of CD44 and CD62L expression. Plots are representative of ≥5 mice/genotype. (**B**) Quantification of splenic CD4^+^CD44^hi^CD62L^lo^ T cells as gated in **A** (*n* ≥ 5, 3- to 4-week-old gDKO and 5- to 6-week-old mice of other genotypes). (**C**) Quantification of splenic FOXP3^–^CD4^+^CD44^hi^CD62L^lo^ T cells as gated in Supplemental Figure 6A from competitive chimeras at indicated time points posttransplant (*n* ≥ 3, pooled from 2 sets of independently generated chimeras). (**D**) Splenocytes from 12 weeks posttransplant DKO:WT = 1:5 chimera were stained to detect anergic T cell subsets. Flow plots depict CD73^hi^FR4^hi^ (anergic) T cells within CD44^lo^CD62L^hi^ (naive), CD44^hi^CD62L^hi^, and CD44^hi^CD62L^lo^ (memory) compartments of CD4^+^FOXP3^–^ cells of each donor genotype. Representative of 7 chimeras, generated in 1 set. (**E**–**G**) Ratio of CD45.2 to CD45.1/2 within CD73^hi^FR4^hi^ gate among naive (**E**), CD44^hi^CD62L^hi^ (**F**), or memory (**G**) CD4^+^ T cell compartments, as gated in **D**. Shown are WT:WT = 1:1 and DKO:WT = 1:5 chimeras at indicated time points posttransplant (*n* ≥ 3 pooled from 2 sets of independently generated chimeras). Ratios were normalized to naive CD4^+^ T cells. Graphs depict mean ± SEM. Statistical significance was assessed by 1-way (**B**) ANOVA with Tukey’s test, 2-way ANOVA with Dunnett’s test (**C**), or 2-tailed unpaired Student’s *t* test with the Holm-Šídák method (**E**–**G**). *P < 0.05; **P < 0.01; ****P < 0.0001. NS, not significant.

gDKO mice exhibited an expanded CD4^+^ T cell effector memory compartment that was not evident in DKO:WT chimeras (Figure 6, A–C). However, CD4^+^ T cell homeostasis is not restored in these chimeras; rather DKO CD4^+^ T cells accumulate in the CD44^hi^ (memory) compartment and upregulate well-established markers of anergy (CD73 and FR4) in a cell-intrinsic manner (Figure 6, D–G, and ref. 39). Similarly, expansion of anergic CD4^+^ T cells was exaggerated in 1:1 DKO:WT chimeras relative to both control chimeras and SKO mice (Supplemental Figure 6, A–H). Moreover, even phenotypically “naive” DKO CD44^lo^CD62L^hi^ CD4^+^ T cells in mixed chimeras upregulated CD73 and FR4, suggestive of antigen encounter (Figure 6, D and E; Supplemental Figure 6, A, C, and F; and ref. 19). Taken together, these data are consistent with escape of self-reactive DKO CD4^+^ T cells from negative selection in the thymus (Figure 2) and acquisition of an anergic phenotype in the periphery.

### Impaired TCR signaling in anergic DKO T cells from competitive chimeras.

**Figure 7:**
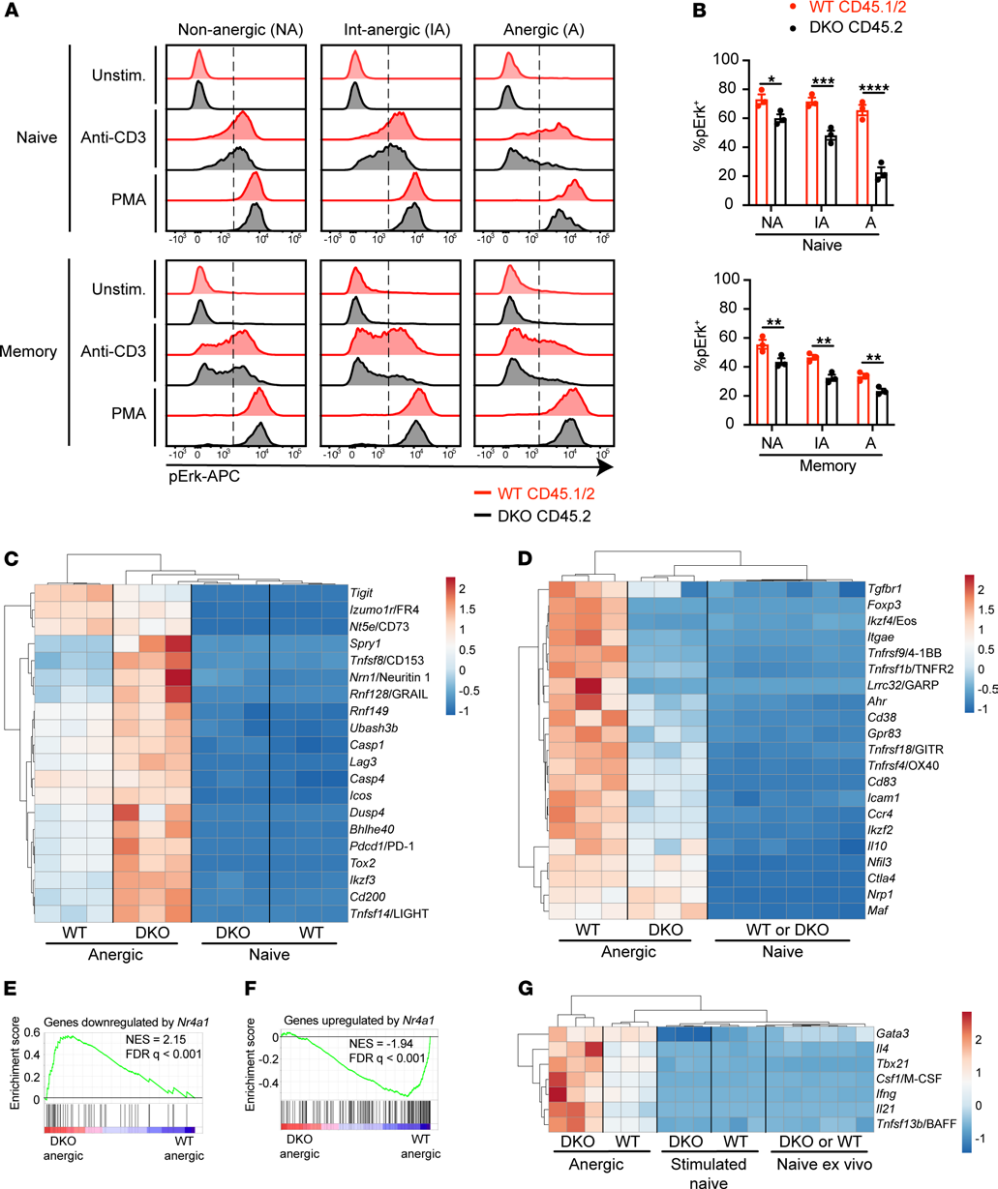
Functional and transcriptional characteristics of anergic CD4^+^ T cells in competitive chimeras. (**A**) Splenocytes from DKO:WT = 1:5 chimera were stimulated with anti-CD3 for 30 seconds followed by secondary cross-linking antibody for 2 minutes, or alternatively with PMA for 2 minutes. Cells were fixed, permeabilized, and then stained to detect surface markers, FOXP3, and phosphorylated Erk (p-Erk). Representative histograms showing intracellular p-Erk expression in nonanergic (CD73^lo^FR4^lo^; NA), intermediate anergic (CD73^int^FR4^int^; IA), or anergic (CD73^hi^FR4^hi^; A) among naive (CD44^lo^CD62L^hi^) or memory (CD44^hi^CD62L^lo^) CD4^+^ T cells gated as in Supplemental Figure 7A. Dashed line shows the threshold of positive gate. Plots are representative of *n* = 6 mice. (**B**) Quantification of %pErk^+^ as in **A** above (*n* = 3 biological replicates, representative of *n* = 2 independent experiments from 1 chimera setup). Graphs depict mean ± SEM. Statistical significance was assessed by 2-tailed unpaired Student’s *t* test with the Holm-Šídák method. *P < 0.05; **P < 0.01; ***P < 0.001; ****P < 0.0001. (**C** and **D**) Naive or anergic CD4^+^ T cells from CD45.1 (WT) or CD45.2 (DKO) cells gated as in Supplemental Figure 7E were sorted directly into buffer RLT for RNA sequencing. ClustVis heatmaps depict expression of selected genes associated with anergy (**C**) or Tregs (**D**). (**E** and **F**) GSEA plots for the genes downregulated (**E**) or upregulated (**F**) by *Nr4a1* (17) against differentially expressed genes (DEGs) in DKO and WT anergic cells. DEGs were defined as genes upregulated in DKO compared with WT anergic cells with *P* < 0.05. NES, normalized enrichment score; FDR, false discovery rate. (**G**) Heatmap shows expression of selected inflammatory mediators.

Canonical functional features of anergic T cells include defective proximal TCR signal transduction and impaired IL-2 production (40, 41). Therefore, we next assessed TCR-induced Erk phosphorylation in T cells from DKO chimeras via a well-established flow-based assay. Since anergic surface markers were largely preserved after TCR stimulation and methanol permeabilization, we could gate cells according to CD73 and FR4 expression and on this basis defined cells as nonanergic, intermediate anergic, or anergic (Supplemental Figure 7A). Consistent with this surface phenotype, we observed progressively impaired TCR-induced Erk phosphorylation of WT memory CD4^+^ T cells across these populations (Figure 7, A and B). Within each gate, DKO CD4^+^ T cells were even more refractory to TCR stimulation than WT cells from the same chimera. Strikingly, unlike cells of WT origin, naive anergic DKO T cells were as refractory as memory anergic T cells. These data suggest that DKO CD4^+^ T cells not only upregulated markers of anergy (Figure 6) but also acquired functional features of anergy and did so to an even greater extent than WT.

Since we observed the accumulation of DKO CD44^hi^CD8^+^ T cells with increased PD-1 expression in DKO:WT chimeras (Figure 5 and Supplemental Figure 5, C and D), we utilized the same approach to assess functional characteristics of DKO CD8^+^ T cells. We found that DKO CD44^hi^CD8^+^ T cells exhibited impaired Erk phosphorylation relative to WT cells within the same chimera (Supplemental Figure 7, B–D).

Of note, Erk phosphorylation downstream of phorbol myristate acetate (PMA) stimulation was intact in both genotypes across all gated populations, suggesting a proximal rather than distal defect in TCR signaling among “tolerant” T cells (Figure 7A and Supplemental Figure 7C). Importantly, TCR-induced Erk phosphorylation was robust in naive/nonanergic DKO T cells (Figure 7, A and B; and Supplemental Figure 7, C and D), implying that defective signal transduction was an acquired feature of tolerant T cells. Collectively, these data suggest that self-reactive DKO T cells escape negative selection and acquire both phenotypic and functional features of antigen experience.

### Upregulation of anergy-associated genes in DKO CD4^+^ T cells.

To define the transcriptome of anergic DKO CD4^+^ T cells using an unbiased approach, we undertook RNA sequencing of DKO and WT cells sorted from 1:5 chimeras. We gated on CD4^+^CD25^–^ cells to exclude a large fraction of FOXP3^+^ Tregs and sorted CD44^hi^CD62L^lo^CD73^hi^FR4^hi^ “anergic” cells (Supplemental Figure 7E). In parallel, we also sorted naive CD4^+^ T cells from each genotype for analysis ex vivo and following 3-hour in vitro TCR stimulation. Since we observed the expansion of anergic cells in the naive CD4^+^ T cell compartment, we gated on CD73^lo^FR4^lo^ within the CD44^lo^CD62L^hi^ gate to collect the least self-reactive naive cells. Indeed, sorted “naive” DKO cells did not express anergy-associated genes ex vivo (Supplemental Figure 7F and Figure 7C). By contrast, “anergic” DKO cells exhibited pronounced upregulation of a subset of anergy-related genes relative to “anergic” WT cells (Figure 7C). This may reflect a high degree of self-reactivity in this compartment due to escape from negative selection. Importantly, although gene expression diverges between DKO and WT anergic cells, much of their transcriptome is shared (Supplemental Figure 7, F and G). These include negative regulators such as Lymphocyte-activation gene 3 (*Lag3*), *Pdcd1/*PD-1, *Rnf128*/GRAIL, and *Spry1* that serve to suppress proximal TCR signaling and may account, at least in part, for the defect in ERK phosphorylation we observed in DKO cells (Figure 7C). Indeed, we supported upregulation of PD-1 and LAG3 expression in DKO anergic cells by flow staining (Supplemental Figure 7, H and I). By contrast, Treg-associated genes such as *Foxp3*, *Ikzf4*/Eos, and *Lrrc32/*GARP are not expressed in anergic DKO cells (Figure 7D), consistent with previous studies revealing an essential role for the NR4A family in induction and maintenance of Treg fate (6, 7, 28, 42). Further validating our data set, gene set enrichment analysis (GSEA) revealed that genes repressed by overexpression of *Nr4a1* (17) are enriched in DKO anergic cells (Figure 7E), and conversely, genes upregulated by Nr4a1 overexpression (17) are enriched in WT anergic cells (Figure 7F) (9, 17). Strikingly, a subset of genes encoding proinflammatory cytokines and mediators was upregulated in anergic DKO cells (Figure 7G), including Th1-related genes (*Tbx21* and *Ifng*) and Th2-related genes (*Gata3* and *Il4*). This is consistent with prior reports identifying a role for the NR4A family in the repression of Th1 and Th2 cell differentiation (7, 42) and may contribute to immune dysregulation observed in DKO:WT chimeras.

### Cell-intrinsic defect in peripheral CD4^+^ DKO T cell tolerance.

**Figure 8:**
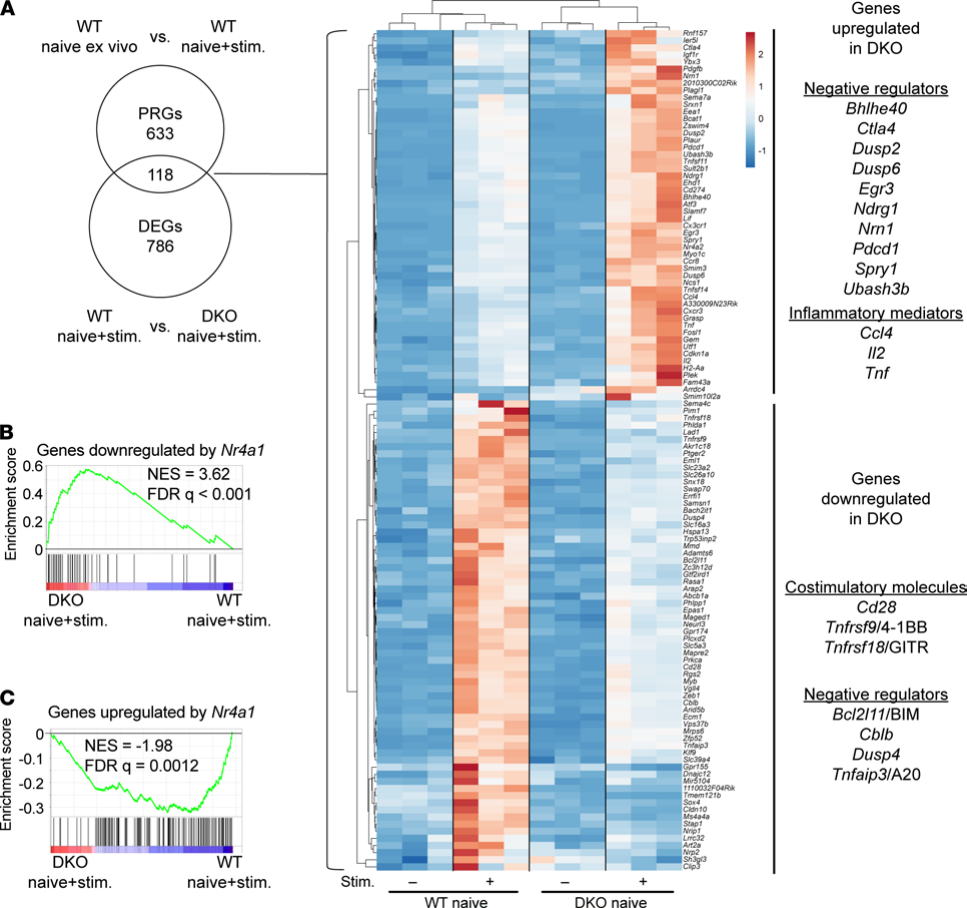
Transcriptional targets of NR4A family in acutely stimulated naive CD4^+^ T cells. (**A**) ClustVis heatmap shows overlap PRGs and DEGs in TCR-stimulated naive CD4^+^ T cells. PRGs defined as genes upregulated in stimulated WT naive CD4^+^ T cells relative to ex vivo with FDR < 0.05, log CPM > 1, and log_2_ fold change > 1.5. DEGs were defined as in Figure 7. (**B** and **C**) GSEA plots for the genes downregulated (**B**) or upregulated (**C**) by *Nr4a1* (17) against DEGs in DKO and WT stimulated naive CD4^+^ T cells.

In order to define how such dysregulated gene expression may arise and to control for self-reactivity of the DKO T cell repertoire, we next sought to identify the immediate targets of the NR4A family in sorted CD73^–^FR4^–^ “naive” CD4^+^ T cells (Supplemental Figure 7E). We compared the expression of primary response genes (PRGs) in sorted naive WT and DKO cells following TCR stimulation (Supplemental Figure 8A). We selected an early 3-hour point to capture peak NR4A protein induction and enrich for direct transcriptional targets (18, 43). Principal component analysis (PCA) segregated DKO from WT cells following acute TCR stimulation, though less robustly than for DKO and WT anergic cells (Supplemental Figure 8B and Supplemental Figure 7G). We focused our attention on PRGs that were differentially expressed between WT and DKO cells (Figure 8A). Among those underinduced in DKO cells, we identified *Bcl2l11*/BIM and negative regulators of TCR signaling including *Cblb*, *Dusp4*, and *Tnfaip3*/A20. Conversely, we observed overinduction of inflammatory mediators such as *Ccl4*, *Il2*, and *Tnf* in DKO cells. We found that genes downregulated by *Nr4a1* overexpression were highly enriched in acutely TCR-stimulated naive DKO cells, while the opposite was true for genes upregulated by *Nr4a1* overexpression (Figure 8, B and C).

**Figure 9:**
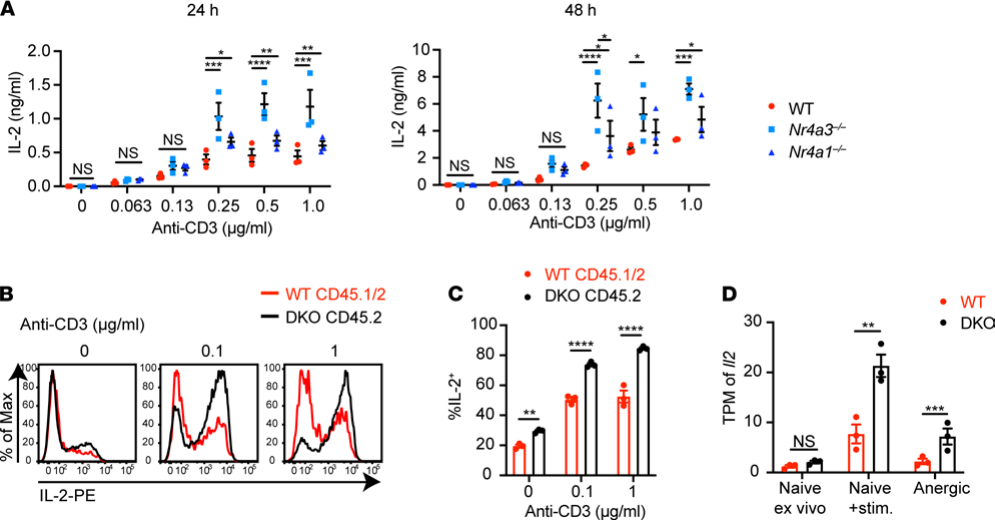
NR4A family negatively regulates IL-2 production in CD4^+^ T cells. (**A**) CD4^+^ T cells were isolated by negative selection from lymph nodes and cultured in plates coated with indicated dose of anti-CD3 + anti-CD28 for 24 hours (left) or 48 hours (right). IL-2 concentration in supernatant was measured with ELISA (*n* = 3 biological replicates). (**B**) Lymph node cells from 10 weeks posttransplant DKO:WT = 1:1 chimera were cultured in plates coated with indicated doses of anti-CD3 for 20 hours. Then cells were restimulated with PMA, ionomycin, and brefeldin for an additional 4 hours. Representative histograms of 3 mice showing intracellular IL-2 in CD4^+^ cells of each donor genotype. (**C**) Quantification of %IL-2^+^ as described for **B** above (*n* = 3 biological replicates from 1 chimera setup). (**D**) Transcripts per million (TPM) of *Il2* detected with RNA sequencing in WT and DKO cells sorted as described. Graphs depict mean ± SEM. Statistical significance was assessed by 2-way ANOVA with Tukey’s test (**A**), 2-tailed unpaired Student’s *t* test with the Holm-Šídák method (**C**), or a paired differential expression analysis with EdgeR comparing samples from the same chimeras (**D**). *P < 0.05; **P < 0.01; ***P < 0.001; ****P < 0.0001. NS, not significant.

Impaired IL-2 production is among the most characteristic features of anergic T cells, while exogenous IL-2 can override anergy in some settings (40, 41), suggesting its dysregulation in DKO T cells may contribute to disruption of T cell tolerance in our chimeras. Indeed, *Il2* has been previously implicated as a target of the NR4A family in the context of anergy and exhaustion (9, 17). We first assessed secreted IL-2 in culture supernatants of *Nr4a1^–/–^* or *Nr4a3^–/–^* SKO CD4^+^ T cells across a broad titration of TCR stimulation (Figure 9A). Both SKO genotypes secrete higher amounts of IL-2 compared with WT, suggesting an additive role for the NR4A family in IL-2 regulation. We next sought to assess IL-2 responses by DKO T cells. To do so, we cultured T cells from DKO:WT chimeras with anti-CD3 and then assessed the capacity for IL-2 production following maximal restimulation with PMA/ionomycin. We observed that, after TCR stimulation, DKO CD4^+^ T cells acquired a much higher capacity for IL-2 production relative to WT, and this was cell intrinsic (Figure 9, B and C). This result was not due to Treg deficiency in the DKO compartment (Supplemental Figure 8C). *Nr4a1^–/–^* or *Nr4a3^–/–^* SKO CD4^+^ T cells each exhibited a less robust but independent increase in capacity for IL-2 production relative to WT (Supplemental Figure 8D). Moreover, *Il2* transcript was upregulated in anergic DKO cells relative to anergic WT cells directly ex vivo (Figure 9D). These data suggest that the role of the NR4A family in restraining IL-2 production is not completely redundant, but rather additive, and affects naive as well as anergic CD4^+^ T cells.

DKO CD8^+^ T cells also exhibited a much higher capacity for IL-2 production than WT cells from the same mixed chimera (Supplemental Figure 8, E and F). Furthermore, CD8^+^ T cells from CD8-cre cDKO mice exhibited a nearly identical phenotype that was more robust than in SKO CD8^+^ T cells from *Nr4a3^–/–^* or CD8-cre *Nr4a1^fl/fl^* mice (Supplemental Figure 8G). These data suggest that the NR4A family negatively regulated the IL-2 locus in peripheral CD8^+^ T cells in a manner that is additive and cell intrinsic, independent of self-reactivity.

### Restoring WT Treg compartment in competitive chimeras alters autoantibody repertoire but does not suppress autoimmunity.

**Figure 10:**
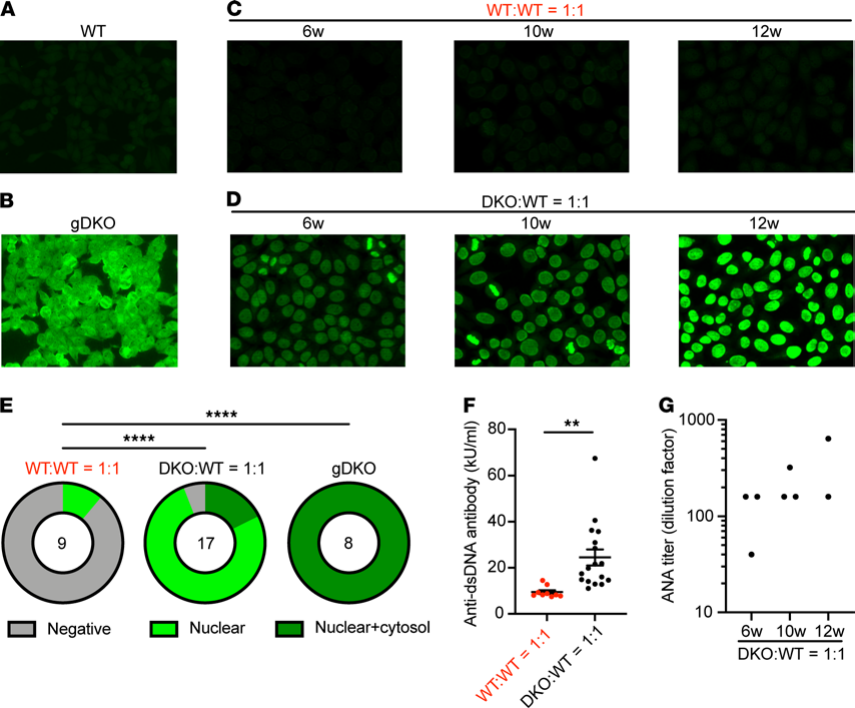
Restoring the Treg compartment in competitive chimeras alters autoantibody repertoire but does not restore tolerance. (**A**–**D**) ANA immunofluorescence images — 1:40 diluted serum of indicated mice was applied to Hep-2 substrate slides, washed, and stained with FITC–anti–mouse IgG. Original magnification, 20×. Images are representative of biological replicates as quantified below (**E** and **G**). (**E**) Graphs depict frequency of negative, nuclear, or nuclear+cytoplasmic Hep-2 cell staining patterns in WT:WT = 1:1 chimera, DKO:WT = 1:1 chimera, and gDKO (1:40 dilution). Data include analysis of serum from 2 sets of independently generated chimeras 6 to 12 weeks posttransplant. Statistical significance was assessed by Fisher’s exact test. ****P < 0.0001. (**F**) Quantification of anti-dsDNA antibody from *n* = 9 WT:WT 1:1 chimeras and *n* = 17 DKO:WT 1:1 chimeras determined by ELISA, pooled from 2 sets of individually generated chimeras. Statistical significance was assessed by 2-tailed unpaired Student’s *t* test. **P < 0.01. (**G**) ANA titer determined with serial 2-fold dilution of serum from chimeras at indicated time points posttransplant stained as in **A**–**D**.

gDKO mice exhibited spontaneous, early-onset development of autoantibodies (Figure 10, A and B). Indirect immunofluorescence assay for autoantibodies revealed both nuclear and cytosolic staining, suggesting a widespread loss of B cell tolerance that occurs with complete penetrance before 4 weeks of age, recapitulating observations in Treg-deficient mice (4). It is possible that this is attributable in part to loss of T follicular regulatory (Tfr) cells in gDKO as seen in other Treg-deficient mice (44). Indeed, we identify a profound cell-intrinsic defect for DKO cells in Tfr (but not T follicular helper, Tfh) compartments in chimeras (Supplemental Figure 9, A and B; and refs. 7, 45). Although older *Nr4a3*^–/–^ (but not *Nr4a1*^–/–^) mice exhibited very low titer autoantibodies with a similar pattern (Supplemental Figure 9, C and D), B cell tolerance was largely preserved in SKO mice. To our surprise, despite reconstitution of the Treg (and Tfr) compartment in DKO:WT chimeras with cells of WT origin, we observed development of high-titer autoantibodies even at early points after reconstitution (Figure 10, C and D). Cytosolic staining by autoantibodies was largely eliminated, but ANAs persisted in both 1:1 and even 1:5 DKO:WT chimeras (Figure 10, E and F; and Supplemental Figure 9, E and F). This suggests that the development of autoimmunity in DKO chimeras was not attributable to a residual or partial Treg defect. The titer of ANAs in DKO:WT chimeras increased with age (Figure 10G and Supplemental Figure 9E). This correlated with progressive accumulation of anergic CD4^+^ T cells, thymic atrophy, and development of polyclonal B cell activation and spontaneous GC expansion, suggestive of evolving immune dysregulation in these chimeras. By contrast, mice in which B cells conditionally lack both *Nr4a1* and *Nr4a3* (mb1-cre cDKO) did not develop ANAs even after 40 weeks (Supplemental Figure 9, G and H). These data imply that ANAs in DKO:WT chimeras are not attributable to a B cell–intrinsic role for the NR4A family. Rather, we propose that — although reconstituting a WT Treg compartment suppresses lethal immune dysregulation in DKO chimeras — tolerance is not fully restored, and this may be due to a profound defect in both negative selection and peripheral T cell tolerance.

## Discussion

A vital and redundant role for NR4A factors in the Treg compartment has made it challenging to isolate and dissect other functions for this family in immune tolerance and homeostasis (6, 7). Unfortunately, conditional genetic strategies alone cannot disentangle requirements for the NR4A family during thymic selection from their obligate function in Tregs. Here we used competitive BM chimeras to reconstitute a functional Treg compartment of WT origin, and this enabled us to unmask additional essential roles for the NR4A family in the preservation of both central and peripheral T cell tolerance under homeostatic conditions.

We confirmed a cell-intrinsic requirement for *Nr4a1* and *Nr4a3* in the Treg compartment as previously reported with CD4-cre conditional DKO and triple-knockout (TKO) mice (6, 7, 28, 42). Concurrently, we also observed expansion of DKO CD25^+^FOXP3^–^ cells both in gDKO mice and DKO chimeras. Sekiya and colleagues propose that this compartment contains highly self-reactive T cells that failed to assume Treg fate and yet escaped censorship by negative selection (28). Formal fate-mapping studies will be important to test this hypothesis. This population of cells may contribute to immune dysregulation in DKO chimeras, but importantly, cells diverted from the Treg fate cannot account numerically for excess DKO single-positive thymocytes that escape negative selection in mixed chimeras, especially since equal or greater advantage for DKO cells relative to WT is observed in CD8SP thymocytes relative to CD4SP.

By contrast, we showed that myeloid cell expansion in gDKO mice is not cell intrinsic because it is almost entirely suppressed in DKO chimeras and is instead likely attributable to loss of Tregs as observed in other Treg-deficient mouse models (2, 3, 37). In support of this hypothesis, CD4-cre TKO mice develop a similar myeloproliferative disorder that is also rescued in competitive chimeras generated with mixtures of CD4-cre TKO and WT donor BM (6). This result emphasizes the need to critically reassess the therapeutic potential of the NR4A family as drug targets in myeloproliferative disorders.

One of the earliest functions identified for the NR4A family is an essential role in antigen-induced cell death and during thymic negative selection, but these studies relied on misexpression of full-length and truncated NR4A-Tg constructs under the control of the proximal Lck promoter (which is active early during the DN stage of thymic development) (13, 21, 22, 33, 34). By contrast, studies of *Nr4a1^–/–^* mice have revealed subtle phenotypes, consistent with redundancy among family members (23, 24, 46). Here we were able to unmask the redundancy between *Nr4a1* and *Nr4a3* during thymic negative selection in a physiological setting for the first time to our knowledge. It is estimated that 6 times more thymocytes are negatively than positively selected in a given time frame (47). Since both 1:1 and 1:5 DKO chimeras harbored 4- to 6-fold more CD4SP and CD8SP DKO cells relative to WT cells when normalized to the preselection DP compartment (Figure 2E), we propose that NR4A-dependent deletion may account for most or all negative selection. Prior studies implicate the NR4A family in negative selection by both ubiquitous and tissue-restricted antigens (TRAs; refs. 22, 24, 48–50). Though our data do not directly distinguish between the two, the striking amplitude of rescue seen in DKO chimeras suggests escape from negative selection by ubiquitous self-antigens (proposed to account for 75% of all deletion) and possibly TRAs as well, but this remains to be determined (47, 51).

Caspase-3 is activated in thymocytes upon TCR stimulation and in the process of negative selection (52). Reduced aCasp3 expression of in vitro stimulated DKO thymocytes suggests caspase-dependent TCR-induced apoptosis is mediated, at least in part, by the NR4A family. BIM/*Bcl2l11*, a member of the Bcl-2 family that can promote Caspase-3 activation, is also essential for thymic negative selection and may represent a transcriptional target for *Nr4a1* (24, 53). Although we find that *Nr4a1* and *Nr4a3* collectively promoted *Bcl2l11* transcription in naive CD4^+^ T cells (Figure 8A), it has also been shown that NUR77 can promote apoptosis by directly binding BCL-2 in the cytosol, inducing a conformational change that exposes its BH3 proapoptotic domain in a manner independent of transcriptional activity of NUR77 (49, 54). It will be important to define which effectors downstream of NR4As mediate negative selection in vivo. It remains to be determined how additional instructional signals modulate NR4A function to promote Treg differentiation or, alternatively, drive deletion of self-reactive thymocytes.

We observe the accumulation of DKO CD44^hi^CD8^+^ T cells in DKO competitive chimeras, and this was eliminated in CD8-cre cDKO mice in which cre-mediated deletion occurred only after thymic selection was complete (Figure 5). We also observed a marked accumulation of CD4^+^ DKO T cells with transcriptional and functional features of anergy (Figure 6 and Figure 7). We propose that these phenotypes reflect escape of self-reactive T cells into the periphery due to a defect in thymic negative selection.

Recent work suggests that *Nr4a1* is required for induction and/or maintenance of CD4^+^ T cell anergy (17); overexpression of *Nr4a1* drives upregulation of a subset of anergy-related genes, whereas deletion of *Nr4a1* prevents generation of functionally tolerant T cells. Similarly, *Nr4a*-TKO chimeric antigen receptor T cells evade exhaustion and eliminate tumors (9). Although DKO T cells acquire features of tolerance in chimeras, we nevertheless observed the development of systemic immune dysregulation and ANAs in DKO chimeras despite reconstitution of a functional Treg compartment, suggesting a residual defect in functional anergy. It remains to be determined if DKO T cells are also resistant to Treg-mediated suppression. We identified upregulation of inflammatory mediators (e.g., *Il2* and *Tnf*) and impaired induction of negative regulators (e.g., *Cblb*, *Tnfaip3*, and *Bcl2l11*/BIM) in naive DKO CD4^+^ T cells following acute TCR stimulation (Figure 8). These and other transcriptional targets of the NR4A family may contribute to impaired peripheral tolerance. Indeed, although suppression of IL-2 production is among the most characteristic features of anergic T cells, we report enhanced capacity for IL-2 production in SKO T cells (consistent with prior studies of *Nr4a1^–/–^* T cells; ref. 17) and much more so in DKO T cells (Figure 9). We suggest this reflects a role for the NR4A family in epigenetic remodeling of the *Il2* locus in response to TCR stimulation. Indeed, NR4A transcription factors modulate chromatin structure in the setting of chronic antigen engagement (9, 17), and interrogation of a recently published ATAC-sequencing data set reveals differentially accessible regions of open chromatin near the *Il2* locus in *Nr4a3^–/–^* CD8^+^ T cells following 12 hours’ TCR stimulation (GSE143513; ref. 55). We propose that self-reactive DKO T cells that have escaped negative selection, Treg differentiation, and peripheral anergy accumulate in the periphery and drive ANA production in DKO chimeras. It remains to be defined whether defective central or peripheral tolerance (or both) is most relevant for the development of autoimmunity in DKO chimeras and whether specific Th subsets (such as Tfh) play a role (42).

Nearly complete redundancy between *Nr4a1* and *Nr4a3* is evident in Tregs and during negative selection; deletion of both family members is necessary to unmask these roles. By contrast, regulation of B cell responses (20) and CD8^+^ T cell exhaustion (9) by the NR4A family appears additive. Based on published work (17) and our observations of the IL-2 module in SKO and DKO T cells, we speculate that regulation of CD4^+^ T cell anergy is similarly additive, but this remains to be fully addressed. Although expression of *Nr4a2* is low in the T cell lineage under steady-state conditions, we also cannot exclude the possibility that *Nr4a2* compensates for and partially masks some immune phenotypes in DKO cells, especially in the context of inflammatory stimuli.

We propose that *Nr4a1* and *Nr4a3* regulate layered T cell tolerance mechanisms to preserve immune homeostasis *under steady-state conditions* (see model, Supplemental Figure 10). In addition, it is likely that NR4A factors also serve to counterregulate inflammatory stimuli and promote a return to homeostasis. Indeed, negative feedback by NR4A restrains responses to LPS in myeloid cells (56) and to antigen stimulation in B cells (20) and suppresses inflammation in immune-mediated disease models (57). Although it remains unclear whether endogenous ligands regulate NR4A function in vivo, small molecule NUR77 agonist (10) and antagonist (11) compounds have been reported. Agonists might be useful to suppress autoimmunity and maintain transplant tolerance. Antagonizing NUR77 and perhaps other NR4A family members could have applications for cancer immunotherapy (9). Since redundancy among NR4A family members is important in both negative selection and in Tregs, selectively targeting individual NR4A family members may allow modulation of antigen-specific T and B cell responses without disrupting global immune homeostasis. Conversely, our studies unmask Treg-independent and redundant roles for *Nr4a1* and *Nr4a3* in maintaining T cell tolerance under homeostatic conditions, with important implications for drug design.

## Methods

### Mice

*Nr4a1^–/–^*, *Nr4a1^fl/fl^*, and *Nr4a3^–/–^* mice were previously described (6, 20, 23). *Nr4a1^fl/fl^* were previously obtained from Catherine Hedrick (La Jolla Institute for Immunology, La Jolla, California, USA) with permission from Pierre Chambon (University of Strasbourg, Strasbourg, France; ref. 6). *Nr4a1^–/–^* mice were obtained from The Jackson Laboratory, and this line is used throughout the manuscript exclusively as single germline knockout comparator (23). *Nr4a3^–/–^* mice were generated in our laboratory as previously described (20). CD8-cre and mb1-cre were obtained from The Jackson Laboratory (38, 58). C57BL/6 mice were from The Jackson Laboratory, and CD45.1^+^ BoyJ mice were from Charles River Laboratories. To generate gDKO *Nr4a1^–/–^ Nr4a3^–/–^* mice, we bred *Nr4a3^–/–^* and *Nr4a1^fl/fl^* mice with germline recombination of the *loxp*-flanked locus and confirmed loss of exon 2 by genomic DNA PCR and transcript quantitative PCR. All strains were fully backcrossed to C57BL/6 genetic background for at least 6 generations. Mice of both sexes were used for experiments between the ages of 3 and 10 weeks except for BM chimeras as described below.

### Antibodies and reagents

#### Antibodies for surface markers.

Antibodies (Abs) against B220, CD3, CD4, CD8, CD11b, CD11c, CD19, CD21, CD23, CD25, CD44, CD45.1, CD45.2, CD62L, CD69, CD73, CD86, CD93 (AA4.1), CD138, CXCR5, Fas, FR4, γδTCR, GL7, Gr1, IgD, MHC-II, NK1.1, PD-1, and pNK conjugated to fluorophores were used (BioLegend, eBiosciences, BD, or Tonbo). See also Supplemental Table 1.

#### Abs for intracellular staining.

FOXP3 Ab conjugated to APC or FITC (clone FJK-16s, Invitrogen), anti-aCasp3 Ab conjugated to APC (clone C92-605, BD Pharmingen), anti-NUR77 conjugated to PE (clone 12.14, Invitrogen), anti–IL-2 Ab conjugated to PE (clone JES6-5H4, Invitrogen), anti–p-ERK (Phospho-p44/42 MAPK [T202/Y204] clone 197G2, Cell Signaling Technology) rabbit Ab, and Goat Anti-Rabbit IgG (H+L) conjugated to APC (Jackson ImmunoResearch) were used. See also Supplemental Table 1.

#### Stimulatory Abs.

Anti-CD3 (clone 2c11) and anti-CD28 (clone 37.51) (BioLegend) and goat anti-Armenian hamster antibody (Jackson ImmunoResearch) were used. See also Supplemental Table 1.

#### ELISA reagents.

High-binding, 96-well, flat-bottom, half-area, clear, polystyrene Costar Assay Plate (Corning), mouse anti-dsDNA IgG–specific ELISA kit (Alpha Diagnostic International), and Mouse IL-2 DuoSet ELISA and DuoSet ELISA Ancillary Reagent Kit 2 (R&D Systems) were used.

#### ANA.

NOVA Lite HEp-2 ANA Substrate Slide and mounting medium (INOVA Diagnostics, Inc, 708100) and FITC Donkey Anti-Mouse IgG (Jackson ImmunoResearch) were used. See also Supplemental Table 1.

#### Culture media.

RPMI-1640 + l-glutamine (Corning/Gibco), Penicillin Streptomycin l-glutamine (Life Technologies), HEPES buffer (10 mM)(Life Technologies), β-mercaptoethanol (55 mM) (Gibco), sodium pyruvate (1 mM) (Life Technologies), nonessential amino acids (Life Technologies), and 10% heat-inactivated FBS (Omega Scientific) were used.

### Flow cytometry

Cells were analyzed on a BD LSRFortessa and sorted on a BD FACSAria. Data analysis was performed using FlowJo (v9.9.6 or v10.7.1) software (BD).

### Intracellular staining to detect aCasp3

Following in vitro stimulation, cells were permeabilized and stained with APC-aCasp3, according to the manufacturer’s protocol (BD Cytofix/Cytoperm kit).

### FOXP3 staining

FOXP3 staining was performed utilizing a FOXP3/transcription factor buffer set (eBioscience) with APC or FITC anti-FOXP3 (clone number FJK-16s), as per manufacturer’s instructions. See also Supplemental Table 1.

### Intracellular staining to detect IL-2

Splenocytes were stimulated with plate-bound anti-CD3 Ab for 20 hours followed by a 4-hour treatment with 20 ng/mL of PMA (MilliporeSigma) and 1 μM of ionomycin (Calbiochem) and protein transport inhibitor cocktail (eBioscience) per manufacturer’s protocol. Following in vitro stimulation, cells were permeabilized and stained, according to the manufacturer’s protocol (BD Cytofix/Cytoperm kit).

### Intracellular staining to detect NUR77

Following 2-hour in vitro stimulation with 20 ng/mL of PMA and 1 μM of ionomycin, cells were fixed in a final concentration of 4% paraformaldehyde for 10 minutes, permeabilized at −20°C with 100% methanol for 30 minutes, and, following washes and rehydration, stained with primary antibody for 60 minutes at 20°C (room temperature).

### LIVE/DEAD staining

LIVE/DEAD Fixable Near-IR Dead Cell Stain kit (Invitrogen) was used. Reagent was reconstituted in DMSO as per manufacturer’s instructions, then diluted 1:1000 in PBS, and cells were stained at a concentration of 1 × 10^6^ cells /100 μL on ice for 15 minutes.

### In vitro T cell culture and stimulation

Flat-bottom, 96-well plates were coated with varying doses of anti-CD3 with or without 2 mg/mL anti-CD28 at 4°C overnight. Splenocytes, lymphocytes, or thymocytes were harvested into single-cell suspension. Splenocytes were subjected to red cell lysis using ACK buffer. Cells were plated at a concentration of 5 × 10^5^ cells/100 μL complete RPMI media (Corning) in antibody-coated, flat-bottom, 96-well plates for varying times.

### BM chimeras

Host mice were irradiated with 2 doses of 5.3 Gy, 4 hours apart, and injected on the same day IV with a total of 2 × 10^6^ donor BM cells at varying ratios (1:1 or 1:5 or without mixture, as noted). Chimeras were sacrificed 6–14 weeks after irradiation for downstream analyses.

### CD4^+^ T cell purification

CD4^+^ T cell purification was performed utilizing magnetic-activated cell sorting separation, per the manufacturer’s instructions. In brief, pooled spleens and/or lymph nodes were prepared utilizing the CD4^+^ T Cell Isolation Kit (Miltenyi Biotec) and purified by negative selection through an LS column (Miltenyi Biotec). Purified CD4^+^ T cells were then subjected to in vitro culture.

### Phospho-flow

Splenocytes were rested at 37°C in serum-free RPMI for 30 minutes. Cells were then stimulated with 10 μg/mL of anti-CD3 (clone 2c11) for 30 seconds followed by 50 μg/mL of anti–Armenian hamster cross-linking antibody for 2 minutes or PMA for 2 minutes. Stimulated cells were fixed with 2% paraformaldehyde and permeabilized with methanol at –20°C overnight. Cells were stained with surface markers and pErk at 20°C.

### RNA sequencing

DKO and WT CD25^–^CD4^+^ T cells were sorted from competitive chimeras to identify either naive (CD44^lo^CD62L^hi^CD73^lo^FR4^lo^) or anergic (CD44^hi^CD62L^lo^CD73^hi^FR4^hi^) populations. Cell populations were sorted directly into RLT + 1% β-mercaptoethanol (BME) buffer. In parallel, sorted naive CD4^+^ T samples were stimulated ex vivo with 8 μg/mL plate-bound anti-CD3 and 2 μg/mL anti-CD28 for 3 hours and lysed in RLT/BME buffer. Libraries were generated by Emory Integrated Genomics Core (EIGC): RNA was isolated using the Quick-RNA MicroPrep kit (Zymo, 11-328M). A total of 2000 cells’ equivalent of RNA was used as input for SMART-seq v4 Ultra Low Input cDNA Synthesis kit (Takara, 634888), and 200 pg of cDNA was used to generate sequencing libraries with the NexteraXT kit (Illumina, FC-121-10300). Libraries were pooled at equimolar ratios and sequenced on the NovaSeq6000 with a PE100 configuration using a NovaSeq 6000 SP Reagent Kit. FASTQ files were trimmed for adapters and low-quality base pairs using Fastp (59), then aligned to mouse genome assembly mm10 using STAR (60). FeatureCounts (61) was used to obtain read count data, and a paired differential expression analysis comparing samples from the same chimeras was performed with edgeR (62). Heatmaps and PCA plots were generated using the ClustVis online tool (https://biit.cs.ut.ee/clustvis/) (63). GSEA was performed with GSEA (v4.1.0) software (UCSD and Broad Institute; ref. 64, 65). FASTQ and TPM data are publicly available (NCBI Gene Expression Omnibus accession number: GSE178782) and Supplemental Data 1 contains analyses.

### ELISA for IL-2 detection

Purified lymph node CD4^+^ T cells were cultured on an anti-CD3/28–coated plate at 1 × 10^5^ cells/well. Plates were spun and supernatants were harvested after 24 hours or 48 hours. IL-2 concentrations in supernatants were measured using a commercial ELISA kit, per the manufacturer’s instructions (R&D Systems). In brief, 96-well plates were coated with 1 μg/mL of capture anti–IL-2 antibody. Supernatants were diluted serially, and IL-2 was detected with detection anti–IL-2 antibody. ELISA plates were developed with a mixture of tetramethylbenzidine and peroxidase, then stopped with 2N sulfuric acid. Absorbance was measured at 450 nm using a spectrophotometer (SpectraMax M5; Molecular Devices).

### ELISA for serum anti-dsDNA

Serum was harvested from blood collected by lateral tail vein sampling or cardiac puncture postmortem. Serum anti-dsDNA titer was measured with a commercial ELISA kit, per the manufacturer’s instructions (Alpha Diagnostic International). In brief, sera were added to plates coated with dsDNA. Anti-dsDNA titer was detected with anti-IgG-HRP. ELISA plates were developed, and absorbance was measured as described above.

### ANA

Serum ANA was detected with NOVA Lite HEp-2 ANA Substrate Slide as per manufacturer’s instructions except for using FITC-conjugated donkey anti-mouse IgG secondary antibody. Images were captured with a Zeiss Axio Imager M2 widefield fluorescence microscope. Images were processed with Zen Pro (Zeiss). To measure titer, serum was serially diluted 2-fold from 1:40 to 1:1280. HEp-2 ANA slides were stained with diluted serum. Images were read by a rheumatologist in a blinded manner, and titer was determined as the detectable lowest dilution of each sample.

### Statistics

Statistical analysis and graphs were generated using Prism v9 (GraphPad Software, Inc). Graphs show mean ± SEM unless otherwise stated. Student’s unpaired or paired *t* test was used to calculate the *P* values for all comparisons of 2 groups, and correction for multiple comparisons across time points or doses was then performed using the Holm-Šídák method. One-way or 2-way ANOVA with follow-up Tukey’s test or Dunnett’s test were performed when more than 2 groups were compared with one another. Fisher’s exact test was used to compare the difference in proportions of 2 groups. Significance was defined as *P < 0.05, **P < 0.01, ***P < 0.001, ****P < 0.0001.

### Study approval

All mice were housed in a specific pathogen–free facility at UCSF according to the university and NIH guidelines. The protocol for use of mice was reviewed and approved by UCSF Institutional Animal Care Use Committee.

## Author contributions

RH, HVN, JLM, and JZ conceived of and designed the experiments. RH, HVN, and JLM performed the experiments. RH, HVN, JLM, RM, and JZ analyzed the data. RH and JZ wrote the manuscript. RH, HVN, JLM, and JZ edited the manuscript.

## Supplementary Material

Supplemental data set 1

Supplemental data set 2

Supplemental table 1

## Version 1 08/03/2021

In-Press Preview

## Version 2 09/08/2021

Electronic publication
