# Supplementary material for: NR4A family members regulate T cell tolerance to preserve immune homeostasis and suppress autoimmunity
Source: JCI Insight. 2021 Sep 8;6(17):e151005. doi: 10.1172/jci.insight.151005 (PMC8492309; doi:10.1172/jci.insight.151005)
Supplement: Supplemental data set 1 [file jciinsight-6-151005-s290.pdf]

Supplementary Figure 1

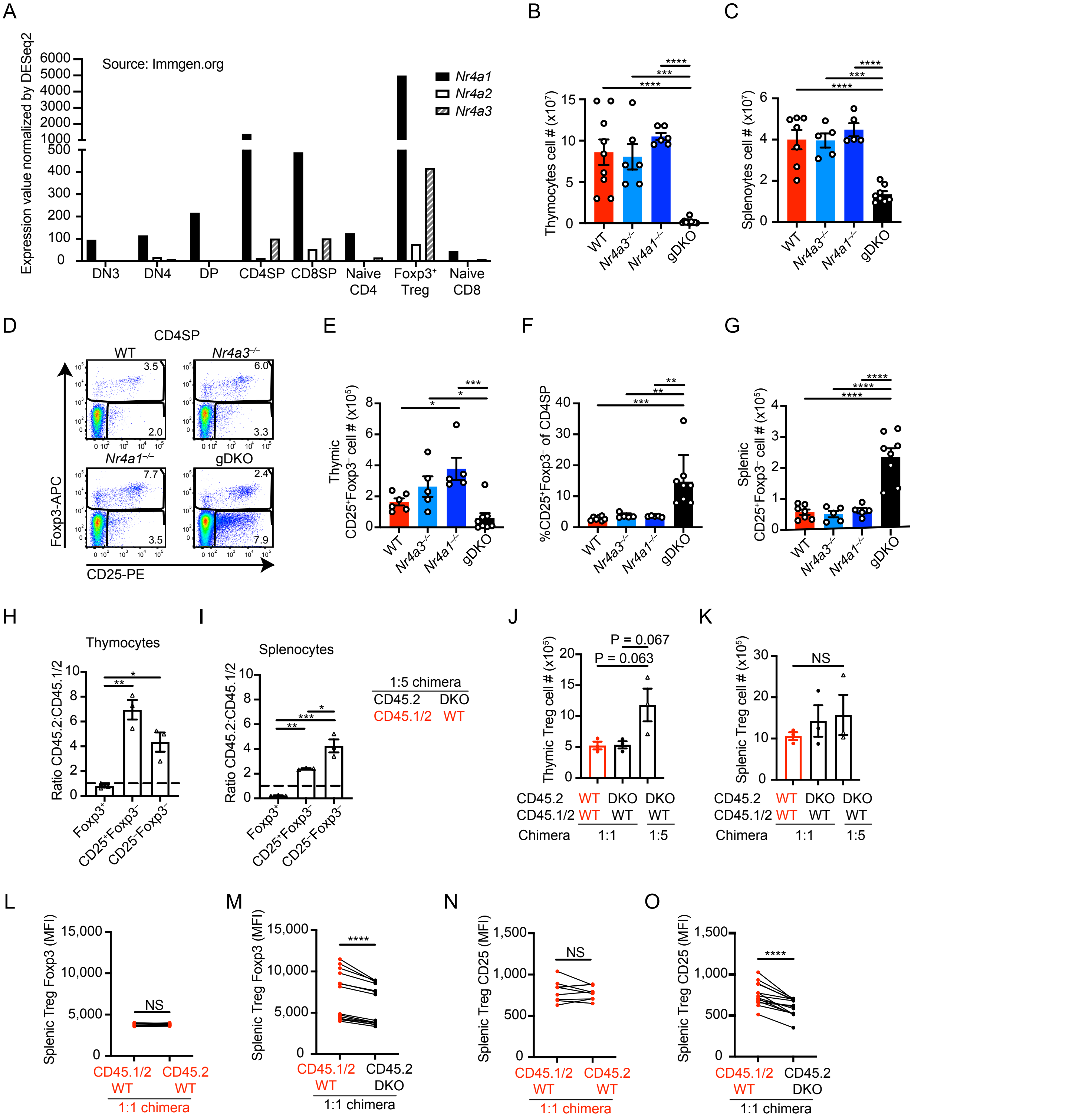

Supplementary Figure 1. Supporting data for Figure 1

A. Expression of *Nr4a* family mRNA in T cell subsets quantified via RNA-seq. Data are exported from Immgen database (http://rstats.immgen.org/Skyline/skyline.html).

B, C. Quantification of viable thymocyte (B) and splenocyte (C) cell number from WT, *Nr4a3*$^{-/-}$, *Nr4a1*$^{-/-}$ and *Nr4a1*$^{-/-}$ *Nr4a3*$^{-/-}$ (gDKO) mice. (Data in C-G include n ≥ 5 biological replicates/genotype, 3 to 4-week-old gDKO and 5 to 6-week-old mice with the other genotypes were analyzed).

D. Representative flow plots showing thymic Treg gate in WT, *Nr4a3*$^{-/-}$, *Nr4a1*$^{-/-}$ and *Nr4a1*$^{-/-}$ *Nr4a3*$^{-/-}$ (gDKO) mice, as determined by FOXP3 and CD25 expression. Plots are representative of at least 5 mice/genotype.

E, F. Quantification of cell number (E) and frequency (F) of thymic CD4SP CD25^{+}FOXP3^{-} cells as gated in (D).

G. Quantification of splenic CD4^{+}CD25^{+}FOXP3^{-} cell number as gated in Fig. 1B (n ≥ 5 biological replicates, 3 to 4-week-old gDKO and 5 to 6-week-old mice with the other genotypes were analyzed).

H, I. Ratio of CD45.2 to CD45.1/2 for Treg, CD25^{+}FOXP3^{-} cells and CD25^{-}FOXP3^{-} cells among thymic CD4SP (H) or splenic CD4^{+} (I) in DKO:WT = 1:5 chimera (n = 3 biological replicates from one chimera setup). Ratios were normalized to DP thymocytes.

J, K. Quantification of thymic (J) and splenic (K) Treg cell number in WT:WT = 1:1, DKO:WT = 1:1 and 1:5 chimera at 6 weeks post-transplant (n = 3 biological replicates pooled from 2 sets of individually generated chimeras).

L-O. Quantification of FOXP3 (L, M) or CD25 (N, O) expression (MFI) on splenic Treg cells of each donor genotype from WT:WT = 1:1 chimeras (L, N) and DKO:WT = 1:1 (M, O) determined via flow staining. Lines connect donor genotypes within an individual chimera (n = at least 6 biological replicates from one chimera setup).Graphs depict mean +/- SEM. Statistical significance was assessed by one-way ANOVA with Tukey's test (B, C, E-K) or two-tailed paired Student's t-test (L-O).

*P < 0.05; **P < 0.01; ***P < 0.001; ****P < 0.0001. NS, not significant.

Supplementary Figure 2

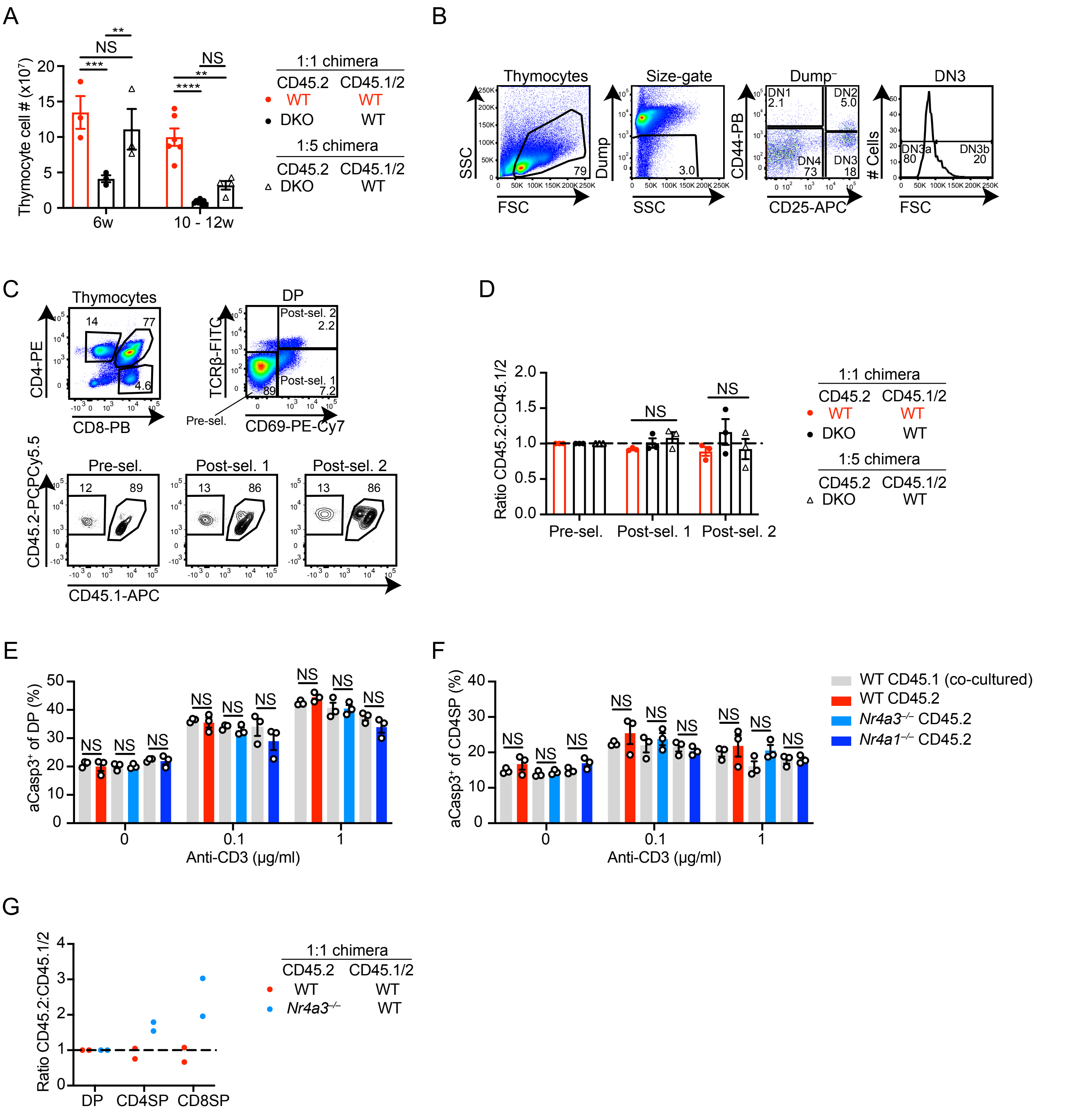

Supplementary Figure 2. Supporting data for Figure 2

A. Quantification of thymocyte cell number in WT:WT = 1:1, DKO:WT = 1:1 and 1:5 chimera at indicated time points post-transplant (n ≥ 3 biological replicates pooled from 3 sets of individually generated chimeras).

B. Representative flow plots show gating strategy to identify pre- and post- b selection subsets DN3a/b in 1:1 DKO:WT chimera. Dump staining includes CD4, CD8, CD3, CD19, γδTCR, NK1.1, pNK, CD11b, Gr1, and CD11c. Plots are representative of 4 chimeras.

C. Representative flow plots show gating strategy to detect pre- and post-positive selection DP subsets in DKO:WT = 1:5 chimera. Plots are representative of 3 chimeras.

D. Ratio of CD45.2 to CD45.1/2 of pre-selection, post-selection 1 and post-selection 2 DP thymocytes (as gated in C) in chimeras, normalized to pre-selection DP (n = 3 biological replicates pooled from 2 sets of individually generated chimeras).

E, F. Thymocytes from CD45.2 WT, *Nr4a3*$^{-/-}$ and *Nr4a1*$^{-/-}$ were mixed with CD45.1 WT thymocytes in 1:1 ratio. Cells were co-cultured, stimulated and stained as described for Figure 2F, I.Quantification of frequency of aCasp3$^+$ cells among DP (E) or CD4SP (F) with indicated dose of anti-CD3 (n = 3 biological replicates).

G. Ratio of CD45.2 to CD45.1/2 thymic subsets in WT:WT = 1:1 chimera and *Nr4a3*$^{-/-}$:WT = 1:1 chimera, normalized to DP thymocytes (n = 2 biological replicates from one chimera setup).

Graphs depict mean +/- SEM. Statistical significance was assessed by two-way ANOVA with Tukey's test (A, D) or two-tailed unpaired Student's t-test with the Holm–Šídák method (E, F). *P < 0.05; **P < 0.01; ***P < 0.001; ****P < 0.0001. NS, not significant.

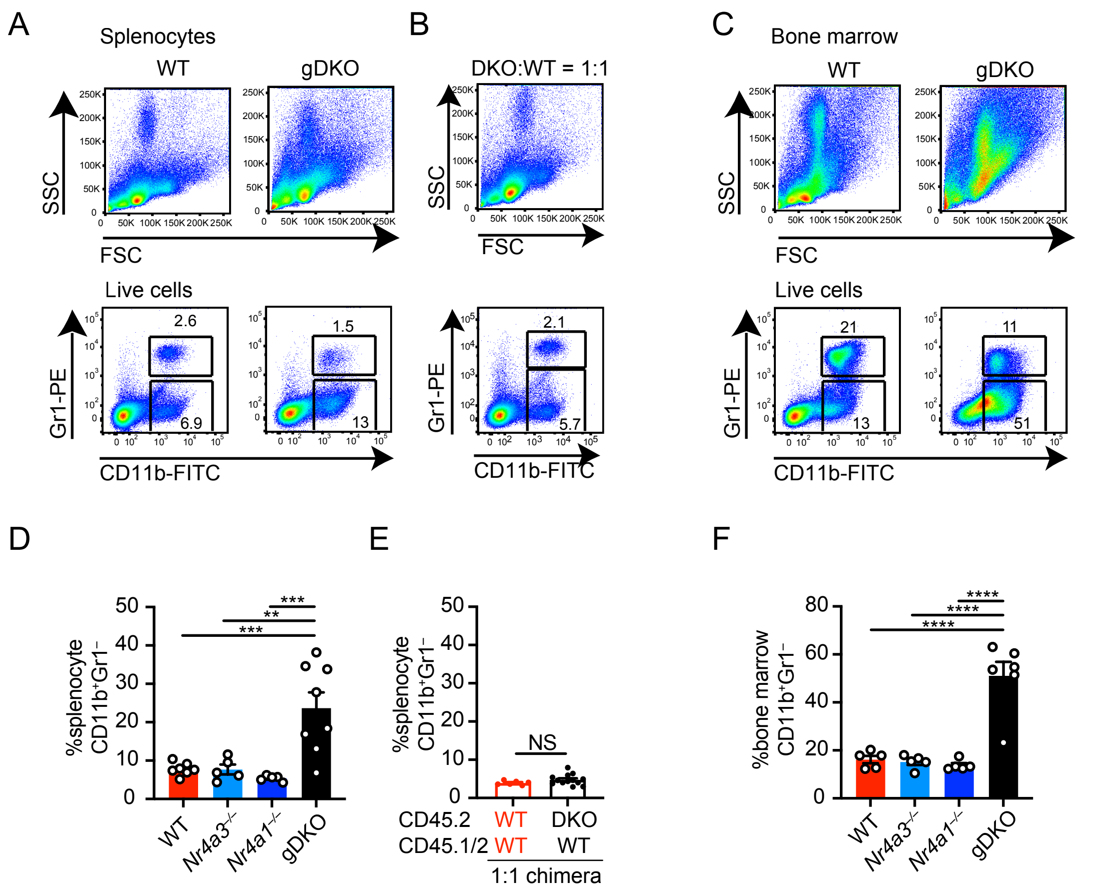

Supplementary Figure 3. Expansion of myeloid cells in spleen and bone marrow of gDKO mice

A-C. Splenocytes (A) and BM cells (C) from WT and gDKO mice, as well as splenocytes from DKO:WT = 1:1 chimera (B) were stained as described for Figure 3A-D. Shown are representative plots of at least 5 mice.

D, F. Quantification of $CD11b^{+}Gr1^{-}$ cell frequency in spleen (D) and bone marrow (F) from WT, *Nr4a3*$^{-/-}$, *Nr4a1*$^{-/-}$ and gDKO mice (n ≥ 5 biological replicates, 3 to 4-week-old gDKO and 5 to 6-week-old mice with the other genotypes were analyzed).

E. Quantification of $CD11b^{+}Gr1^{-}$ cell frequency in spleen from WT:WT = 1:1 and DKO:WT = 1:1 chimera (n ≥ 3 biological replicates pooled from 2 sets of individually generated chimeras).

Graphs depict mean +/- SEM. Statistical significance was assessed by one-way ANOVA with Tukey's test (D, F) or two-tailed unpaired Student's t-test (E). **P < 0.01; ***P < 0.001; ****P < 0.0001. NS, not significant.

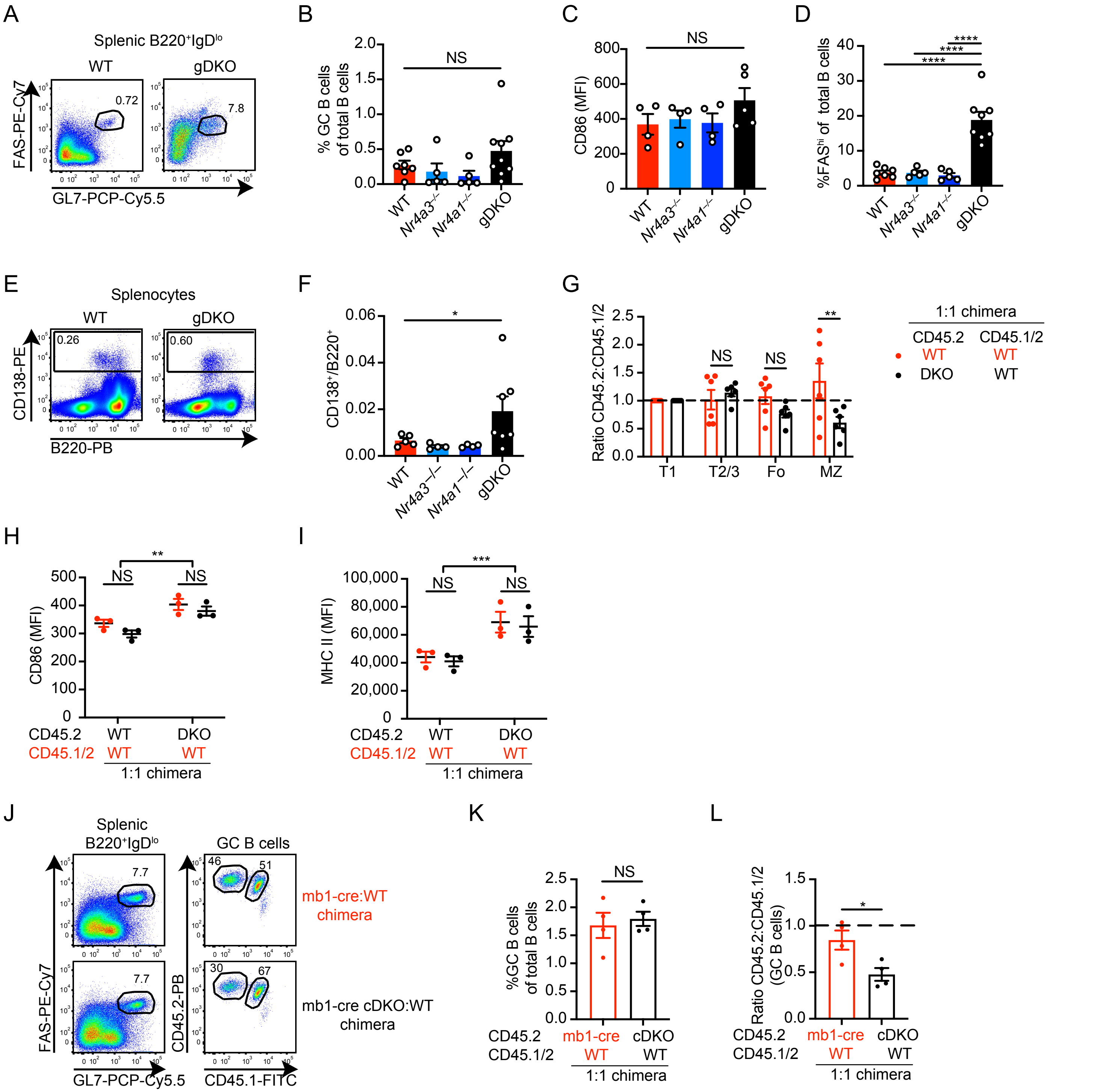

Supplementary Figure 4. Supporting data for Figure 4

A. Representative flow plots show FAS^hi GL7^+ GC B cells from WT and gDKO mice as described for Figure 4D. Plots are representative of 6 mice.

B. Quantification of frequency of GC B cells, as gated in (A) above, among total B cells from WT, *Nr4a3*−/−, *Nr4a1*−/− and gDKO mice (n ≥ 5 biological replicates).

C. Quantification of CD86 MFI on splenic B cells from WT, *Nr4a3*−/−, *Nr4a1*−/− and gDKO mice (n ≥ 4 biological replicates).

D. Quantification of FAS^hi cells among B220^+ cells in spleen from WT, *Nr4a3*−/−, *Nr4a1*−/− and gDKO mice (n ≥ 5 biological replicates).

E. Representative flow plots show CD138^+ cells in splenocytes from WT and gDKO mice. Plots are representative of 4 mice.

F. Quantification of ratio of CD138^+ to B220^+ cells, as gated in (E) above, from WT, *Nr4a3*−/−, *Nr4a1*−/− and gDKO mice (n ≥ 4 biological replicates).

3 to 4-week-old gDKO and 5 to 6-week-old mice with the other genotypes were analyzed (B, C, D, F).

G. The ratio of CD45.2 to CD45.1/2 for transitional1 (T1), T2/3, follicular (Fo) and marginal zone (MZ) B cell subsets (gated on the basis of B220, CD21, CD23, and CD93) from WT:WT and DKO:WT = 1:1 chimera, normalized to T1 subset (n = 6 biological replicates from one chimera setup).

H, I. Quantification of CD86 (H) and MHC II (I) MFI on splenic B cells of each donor genotype in in WT:WT and DKO:WT = 1:1 chimera (n = 3 biological replicates from one chimera setup).

J. Representative flow plots showing FAS^hi GL7^+ GC B cells in spleen from mb1-cre:WT = 1:1 chimera and mb1-cre *Nr4a1*^fl/fl *Nr4a3*−/− (cDKO):WT = 1:1 chimera on the left. Right-hand plots depict CD45.2 cDKO donor and CD45.1/2 WT donor populations. Plots are representative of n = 4 biological replicates.

K. Quantification of frequency of GC B cells among total B cells as described for J above.

L. Ratio of CD45.2 to CD45.1/2 GC B cells in spleen from chimeras described in (J) above, normalized to B220^+IgD^hi cells (data in K, L represent n = 4 biological replicates from n = 2 independent experiments with one chimera setup).

Graphs depict mean +/- SEM. Statistical significance was assessed by Brown-Forsythe ANOVA (B, F), one-way ANOVA with Tukey's test (C, D) or two-tailed unpaired Student's t-test with (G, H, I) or without (K, L) the Holm–Šídák method. *P < 0.05; **P < 0.01; ***P < 0.001; ****P < 0.0001. NS, not significant.

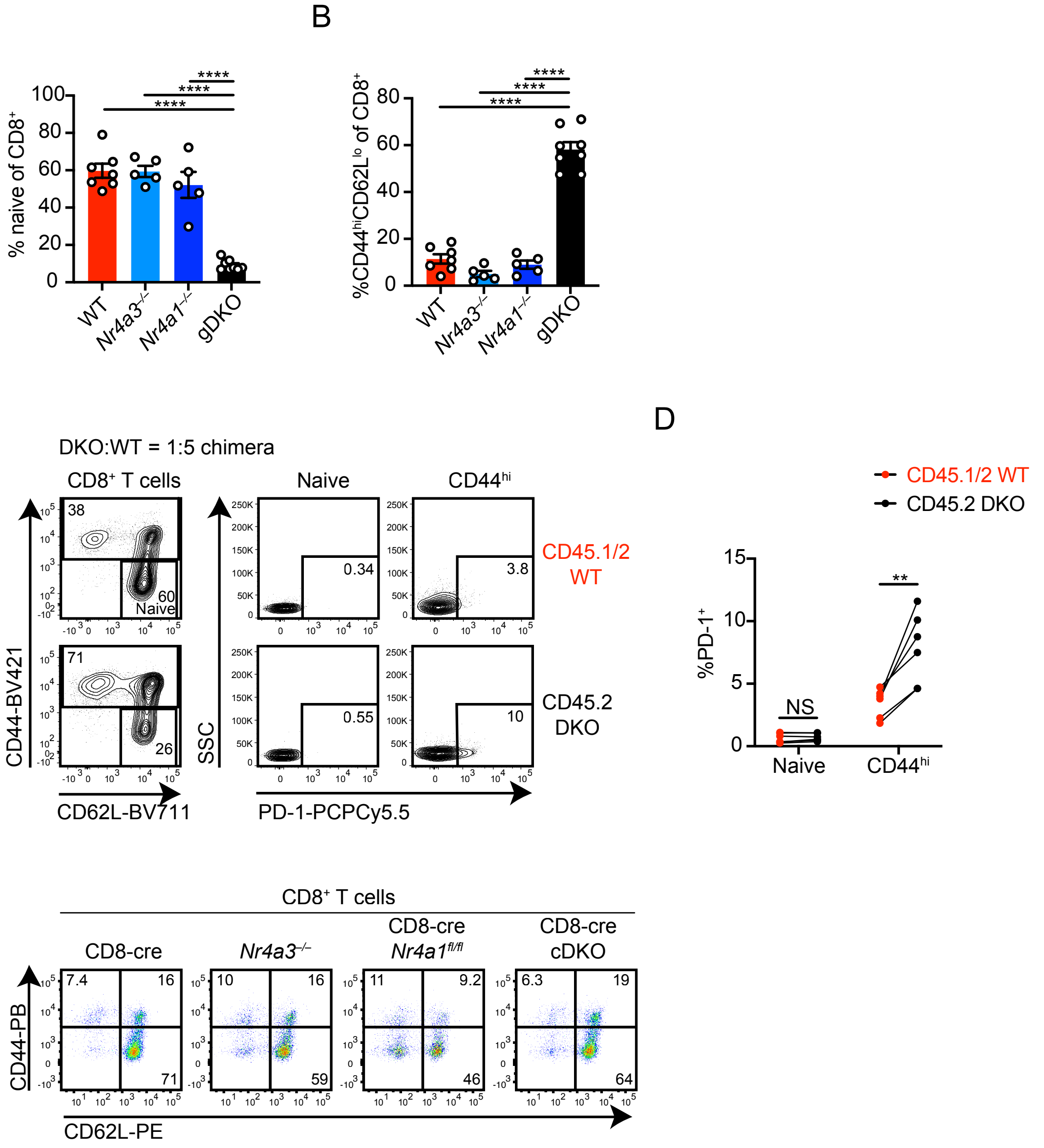

Supplementary Figure 5. Supporting data for Figure 5

A, B. Quantification of frequency of CD8⁺ naive CD44loCD62Lhi (A) and CD44hiCD62Llo (B) cells from WT, *Nr4a3*–/–, *Nr4a1*–/– and gDKO mice as gated in Fig. 5A (n ≥ 5 biological replicates, 3 to 4-week-old gDKO and 5 to 6-week-old mice with the other genotypes were analyzed).

C. Splenocytes from DKO:WT = 1:5 chimera was stained to detect PD-1 expression on naive CD44loCD62Lhi and on CD44hi CD8⁺ cells among each donor genotype. Plots are representative of 6 mice.

D. Quantification of %PD-1⁺ of naive and CD44hi CD8⁺ cells as gated in C above. Lines connect donor genotypes within an individual chimera (n = 6 biological replicates from one chimera setup).

E. Splenocytes from CD8-cre, *Nr4a3*–/–, CD8-cre *Nr4a1*fl/fl and CD8-cre *Nr4a1*fl/fl *Nr4a3*–/– (cDKO) mice were stained as described for Fig. 5A to detect CD8⁺ T cell subsets. Plots are representative of 3 mice and correspond to quantification in Fig. 5H.

Graphs depict mean +/- SEM. Statistical significance was assessed by one-way ANOVA with Tukey's test (A, B) or two-tailed unpaired Student's t-test with the Holm–Šídák method (D). **P < 0.01; ****P < 0.0001. NS, not significant.

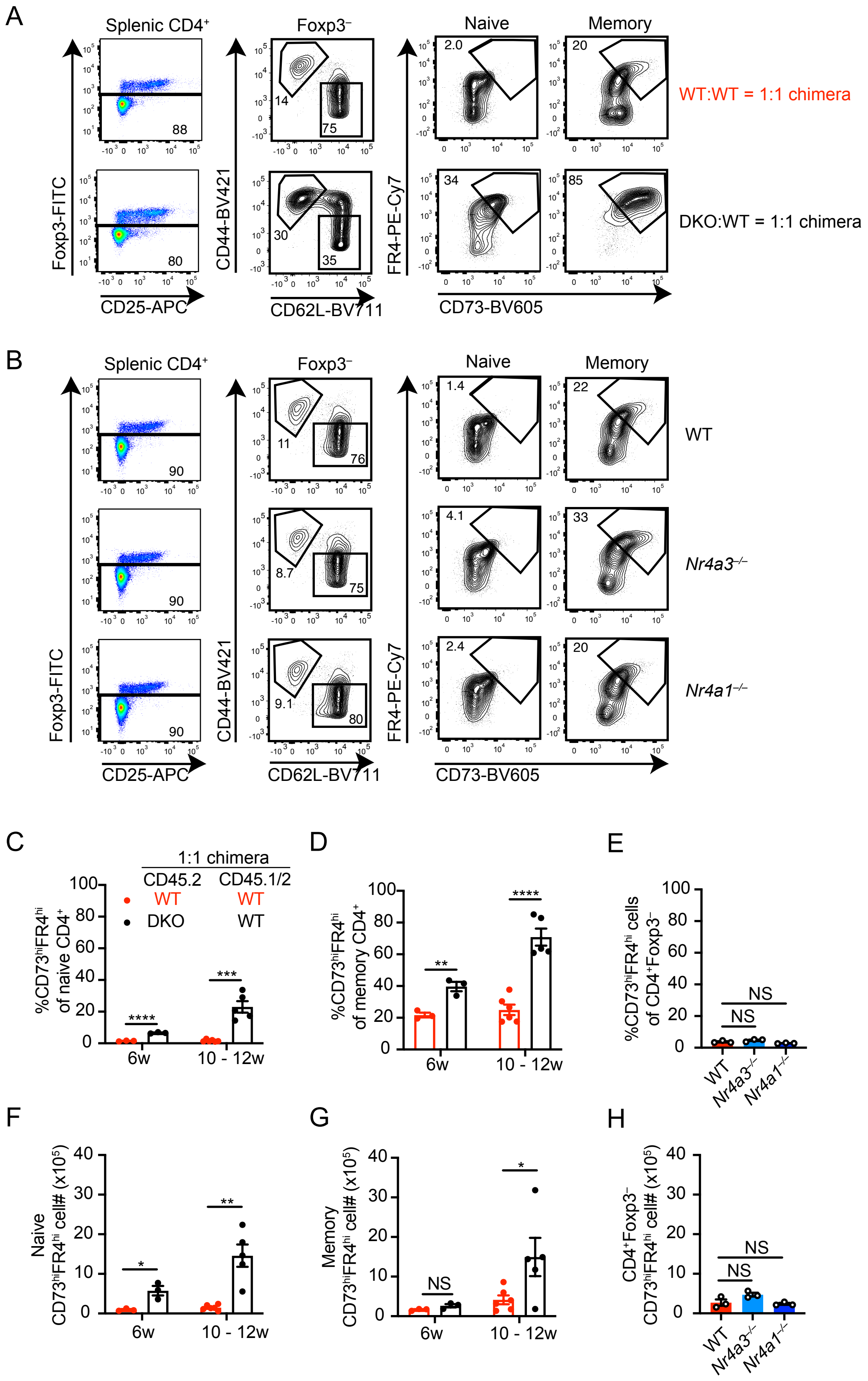

Supplementary Figure 6. Supporting data for Figure 6

A. Splenocytes from 10 weeks post-transplant WT:WT = 1:1 and DKO:WT = 1:1 chimeras were stained as described for Fig. 6D to identify CD73^{hi}FR4^{hi} (anergic) T cells. Plots are representative of 3 mice.

B. Splenocytes from WT, *Nr4a3*–/– and *Nr4a1*–/– mice were stained as described for Fig. 6D. Plots are representative of 3 mice.

C, D, F, G. Quantification of frequency (C, D) and cell number (F, G) of CD73^{hi}FR4^{hi} T cells among naive (C, F) or memory (D, G) CD4^{+} cells in WT:WT = 1:1 chimera and DKO:WT = 1:1 chimera at indicated time points post-transplant (n ≥ 3 biological replicates from one chimera setup).

E, H. Quantification of frequency (E) and cell number (H) of CD73^{hi}FR4^{hi} T cells among total CD4^{+}FOXP3$^{–}$ T cells from WT, *Nr4a3*–/– and *Nr4a1*–/– mice (n = 3 biological replicates).

Graphs depict mean +/- SEM. Statistical significance was assessed by two-tailed unpaired Student's t-test with the Holm–Šídák method (C, D, F, G) or one-way ANOVA with Dunnett's test (E, H). *P < 0.05; **P < 0.01; ***P < 0.001; ****P < 0.0001. NS, not significant.

Supplementary Figure 7

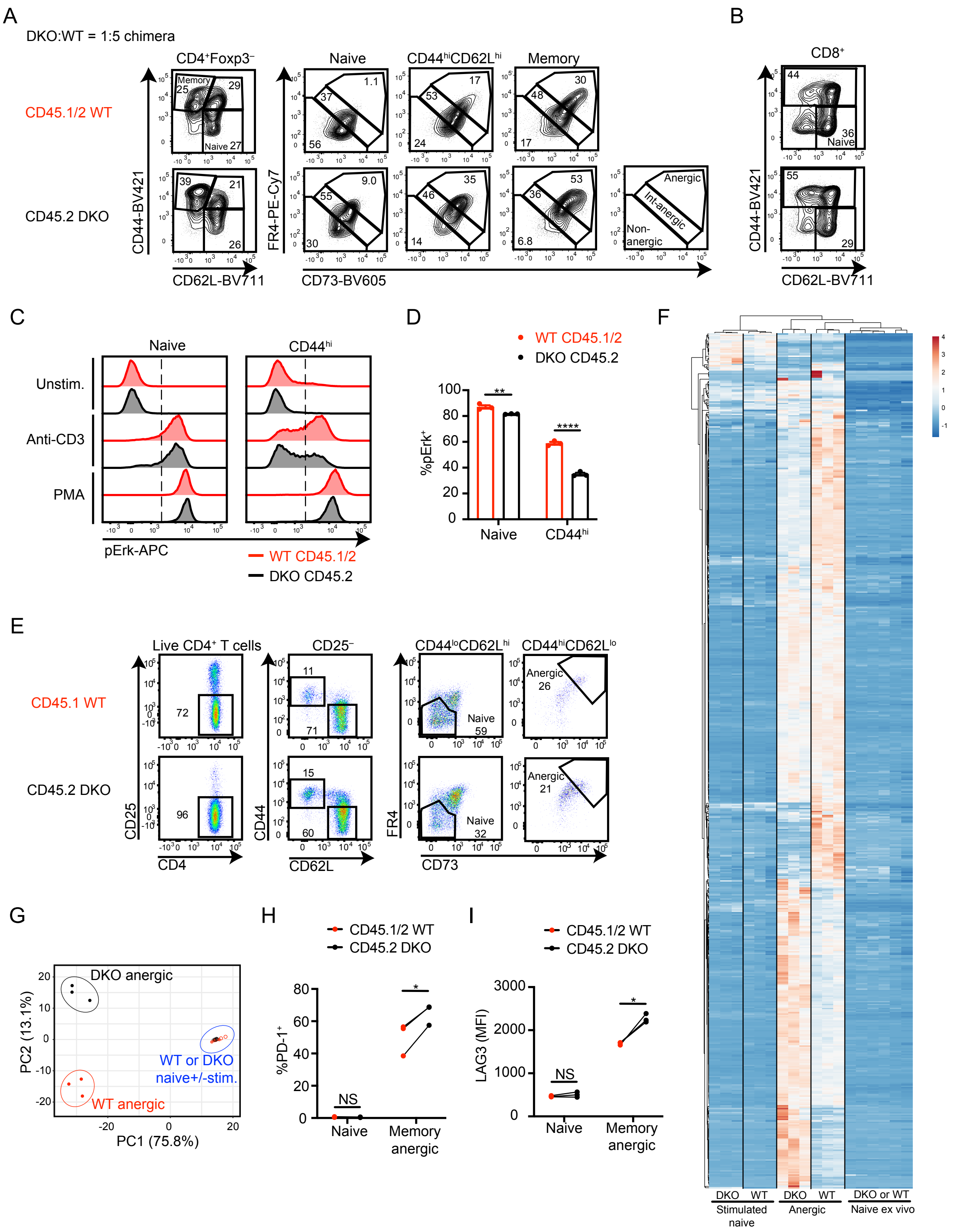

Supplementary Figure 7. Supporting data for Figure 7

A. Gating strategy corresponding to Fig. 7A to identify splenic CD4⁺ T cell sub-populations for phosflow assay. Plots are representative of 3 mice.

B, C. Splenocytes from DKO:WT = 1:5 chimera were stimulated and stained as described in Fig. 7A. (B) Representative plots show naive and CD44^{hi}CD8^{+} T cell gates. (C) Representative histograms show intracellular pErk expression in naive and CD44^{hi}CD8^{+} T cells. Vertical dashed line shows the threshold of positive gate. Plots are representative of 6 mice.

D. Quantification of %pErk^{+} as described in C above (n = 3 biological replicates, representative of n = 2 independent experiments from one chimera setup).

E. Gating strategy for sorting naive and anergic CD4^{+} T cells from competitive chimeras. Splenocytes and lymph node cells from DKO:WT = 1:5 chimera were pooled, and CD4^{+} T cells were negatively isolated with MACS. Enriched CD4^{+} T cells were stained and pre-gated on live CD4^{+}CD45.1^{+} (WT) or live CD4^{+}CD45.2^{+} (DKO). CD25^{-}CD44^{lo}CD62L^{hi}CD73^{lo}FR4^{lo} cells were sorted as “naive”, and CD25^{-}CD44^{hi}CD62L^{lo}CD73^{hi}FR4^{hi} cells were sorted as “anergic”.

F. Heatmap showing expression levels of anergy-related genes in each subset detected with RNA-seq as described in Fig. 7. Anergy-associated genes were identified as genes upregulated in WT anergic cells relative to WT naive cells identified with FDR < 0.05, CPM > 2, and fold change > 4.

G. Principal component analysis (PCA) plot of sorted cells assessed for the expression of anergy-related genes as described for F above. F, G.

H, I. Splenocytes from DKO:WT = 1:5 chimera 12 (I) or 13 weeks (H) post-transplant were stained to detect PD-1 expression on naive (FOXP3^{-}CD44^{lo}CD62L^{hi}CD73^{lo}FR4^{lo}) and memory anergic (FOXP3^{-}CD44^{hi}CD62L^{lo}CD73^{hi}FR4^{hi}) CD4^{+} cells of each donor genotype. Shown are quantification of %PD-1^{+} (H) or MFI of LAG3 (I) of naive and memory anergic. Lines connect donor genotypes within an individual chimera (n = 3 biological replicates from one chimera setup). Graphs depict mean +/- SEM. Statistical significance was assessed by two-tailed unpaired (D) or paired (H, I) Student’s t-test with the Holm–Šídák method. *P < 0.05; **P < 0.01; ****P < 0.0001. NS, not significant.

Supplementary Figure 8

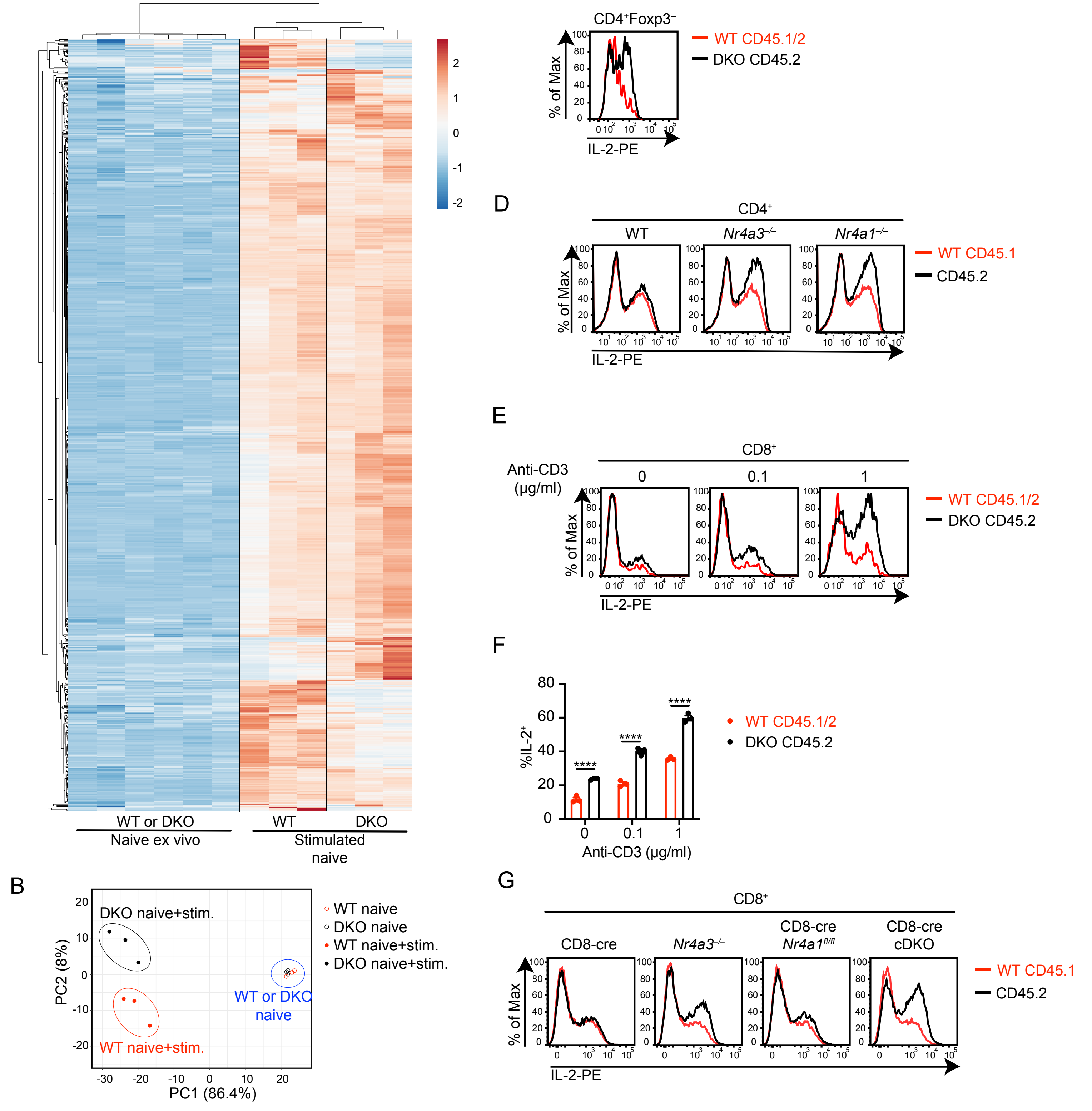

Supplementary Figure 8. Supporting data for Figure 8

A. Heatmap showing expression levels of all primary response genes (PRGs) in sorted naive CD4$^+$ T cells with or without TCR stimulation in vitro. PRGs were defined as described in Fig. 8A.

B. Principal component analysis (PCA) plot of naive CD4$^+$ T cells with or without TCR stimulation in vitro assessed for the expression of PRGs as described for A above. Heatmap and PCA plot were generated using the ClustVis online tool (https://biit.cs.ut.ee/clustvis/).

C. Lymph node cells from DKO:WT = 1:1 chimera were cultured with 0.1 μg/ml of plate-bound anti-CD3 and treated as described for Fig. 8E. Cell surface was stained with CD4, CD8, CD45.1 and CD45.2, followed by permeabilization and intracellular staining for FOXP3 and IL-2. Shown is a representative histogram pre-gated on CD4$^+$FOXP3$^-$ of each genotype.

D. Lymph node cells from WT, *Nr4a3*$^{-/-}$ and *Nr4a1*$^{-/-}$ mice were mixed with CD45.1 lymph node cells, then cultured with 0.1 μg/ml of plate-bound anti-CD3 and stained as described for Fig. 8E. Plots are representative of 3 mice from one chimera setup.

E, F. Lymph node cells from 10 weeks post-transplant DKO:WT = 1:1 chimera were stimulated and stained as described in Fig. 8E. (E) Representative histograms show intracellular IL-2 in CD8$^+$ T cells of each genotype. (F) Quantification of %IL-2$^+$ in CD8$^+$ T cells (data in E, F represent n = 3 biological replicates from one chimera setup). Graphs depict mean +/- SEM. Statistical significance was assessed by two-tailed unpaired Student's t-test with the Holm–Šídák method. ****$P < 0.0001$.

G. Lymph node cells from CD8-cre, *Nr4a3*$^{-/-}$, CD8-cre *Nr4a1*$^{fl/fl}$ or CD8-cre *Nr4a1*$^{fl/fl}$ *Nr4a3*$^{-/-}$ (cDKO) were mixed with CD45.1 lymph node cells, cultured with 1 μg/ml of plate-bound anti-CD3, and then treated and stained as described for Fig. 8E. Representative histograms show intracellular IL-2 in CD8$^+$ T cells of each genotype. Plots are representative of 2 independent experiments.

Supplementary Figure 9

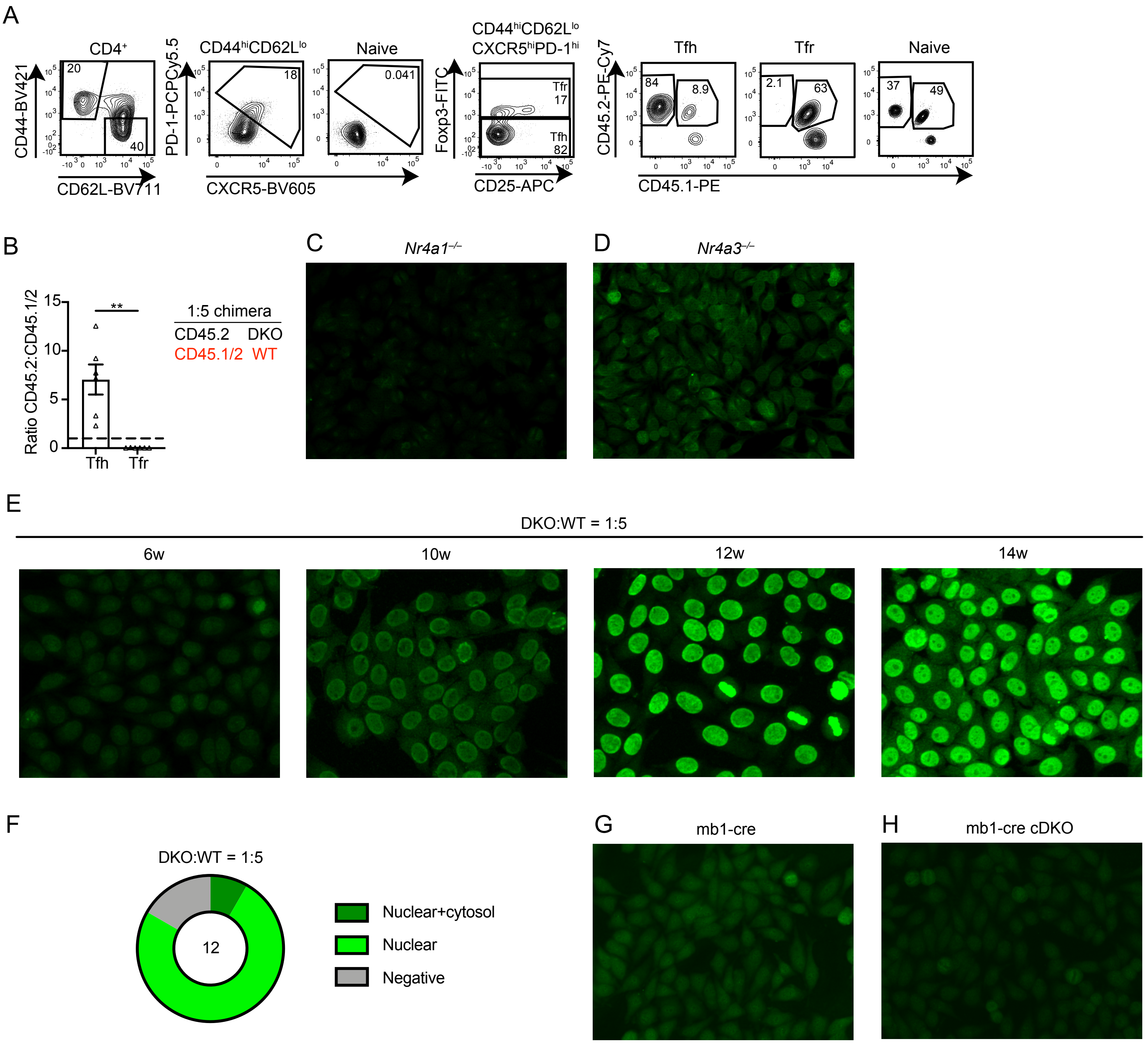

Supplementary Figure 9. Supporting data for Figure 9

A. Splenocytes from DKO:WT = 1:5 chimera were harvested and stained with CD4, CD25, CD44, CD62L, CXCR5 and PD-1, then permeabilized and stained with FOXP3. Representative plots show FOXP3^{-} follicular helper T cells (Tfh) and FOXP3^{+} follicular regulatory T cells (Tfr) within CD4^{+}CD44^{hi}CD62L^{lo}CXCR5^{hi}PD-1^{hi} gate. Naive CD4^{+} T cell gate is shown for reference to define Tfh/Tfr gate. Donor genotype gates among each population are shown.

B. Ratio of CD45.2 to CD45.1/2 for Tfh or Tfr, normalized to naive CD4^{+} as gated in A above (n = 3 biological replicates from one chimera setup). Statistical significance was assessed by two-tailed unpaired Student's t-test. **P < 0.01.

C, D, E, G, H. Anti-nuclear antibody (ANA) immunofluorescence images as described for Fig. 9A-D. Serum was collected from 12-week-old *Nr4a1*$^{-/-}$ (C), *Nr4a3*$^{-/-}$ (D) mice, from DKO:WT = 1:5 chimera at 6, 10, 12, or 14 weeks post-transplant (E), and from mb1-cre *Nr4a1*$^{fl/fl}$ *Nr4a3*$^{-/-}$ (mb1cre-cDKO) (G) or mb1-cre (H) non-competitive chimeras 40 weeks post-transplant. Images are representative of n = 3 (mb1-cre chimera and DKO:WT = 1:5 chimera), n = 5 (mb1-cre cDKO chimera) or n = 10 (*Nr4a1*$^{-/-}$ and *Nr4a3*$^{-/-}$) biological replicates.

F. Graph depict frequency of Hep-2 staining patterns in DKO:WT = 1:5 chimera from one chimera setup.

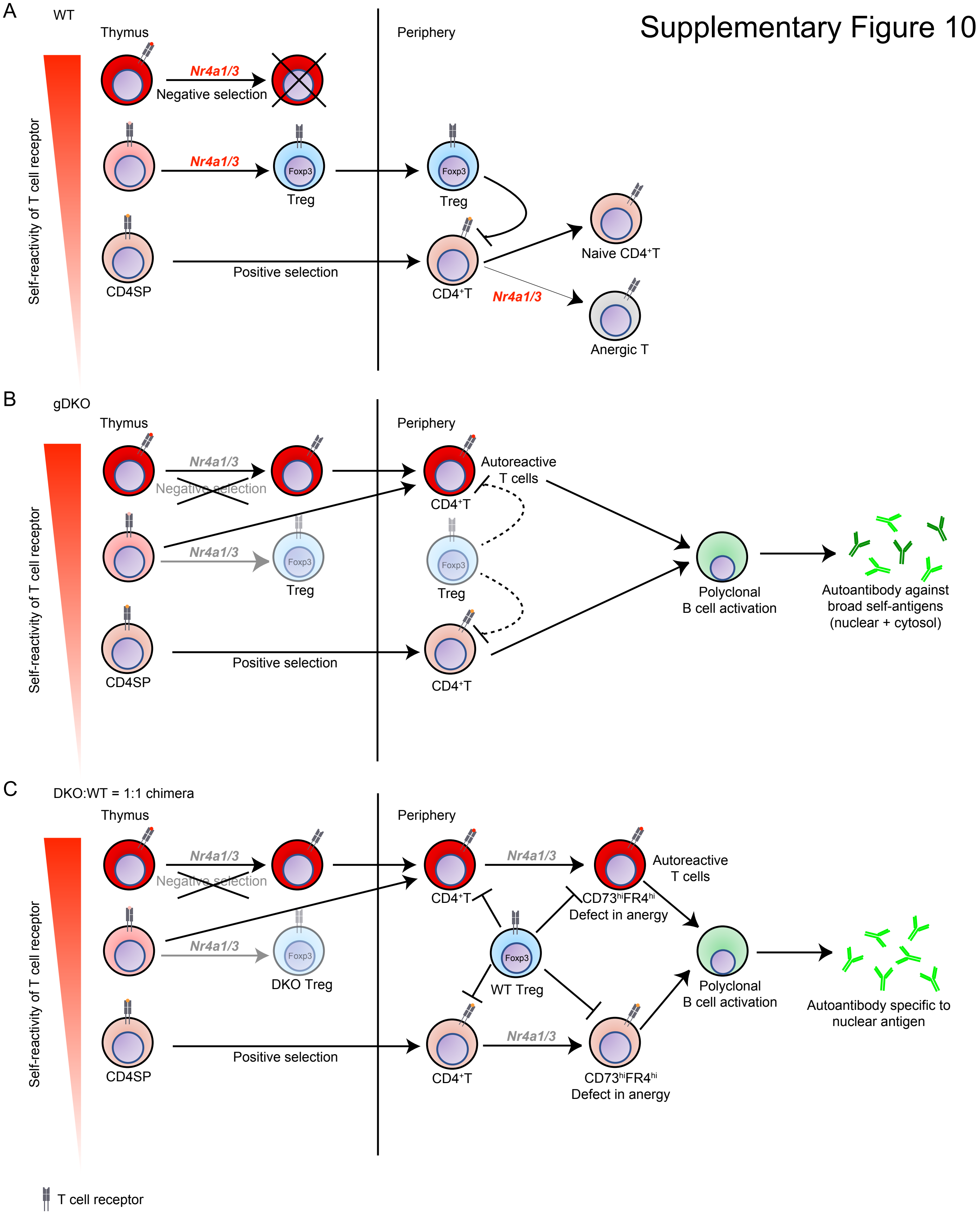

Supplementary Figure 10. Model. Roles for NR4A family in T cell tolerance and immune homeostasis

A. In WT, central and peripheral tolerance are intact. *Nr4a1* and *Nr4a3* mediate thymic negative selection and Treg homeostasis. *Nr4a1* and *Nr4a3* also play a role on induction and maintenance of anergy in periphery.

B. In *Nr4a1*$^{-/-}$ *Nr4a3*$^{-/-}$ (gDKO) mice, both thymic negative selection and Treg homeostasis are impaired, and highly autoreactive T cells escape to periphery. Autoreactive CD4^{+} T cells are activated in part because of Treg deficiency. Polyclonal B cells are activated by autoreactive T cells and produce autoantibody against a broad spectrum of self-antigens as detected by both cytoplasmic and nuclear Hep-2 staining pattern.

C. Treg compartment is reconstituted by WT donor BM in DKO:WT competitive chimeras. However, negative selection of self-reactive DKO thymocytes is still impaired. Self-reactive DKO T cells that escaped negative selection encounter self-antigens in the periphery, acquire features of anergy including expression of CD73 and FR4, yet exhibit a defect in peripheral tolerance. WT-origin Treg and cell-intrinsic peripheral tolerance mechanisms are insufficient to completely suppress autoreactive DKO T cells, which in turn drive anti-nuclear autoantibody production. Although Treg compartment restores some immune homeostasis (and tolerance to cytosolic antigens as detected by Hep-2 staining), autoimmunity is not suppressed due to role of NR4A family in other T cell-intrinsic tolerance mechanisms.
