# Supplementary material for: NR4A family members regulate T cell tolerance to preserve immune homeostasis and suppress autoimmunity
Source: JCI Insight. 2021 Sep 8;6(17):e151005. doi: 10.1172/jci.insight.151005 (PMC8492309; doi:10.1172/jci.insight.151005)
Supplement: Supplemental table 1 [file jciinsight-6-151005-s292.pdf]

Antibodies for surface markers:

Antibody	Clone	Vendor	Catalog number
Rat Anti-Mouse CD45R (B220) Pacific Blue	RA3-6B2	BD Pharmingen	558108
Rat Anti-Mouse CD45R (B220) Pacific Blue	RA3-6B2	Tonbo Biosciences	65-0452-U100
Rat Anti-Mouse CD3e PE	145-2C11	Tonbo Biosciences	50-0031-U100
Rat Anti-Mouse CD8a PerCPCy5.5	GK1.5	Southern Biotech	1540-09L
Rat Anti-Mouse CD4 BUV395	GK1.5	BD Pharmingen	563790
Rat Anti-Mouse CD8a Pacific Blue	53-6.7	BD Pharmingen	558106
Rat Anti-Mouse CD8a PECy7	53-6.7	Tonbo Biosciences	60-0081
Rat Anti-Mouse CD8a PerCPCy5.5	53-6.7	Tonbo Biosciences	65-1886-U100
Rat Anti-Human/Mouse CD11b FITC	M1/70	Tonbo Biosciences	35-0112
Rat Anti-Human/Mouse CD11b PE	M1/70	BD biosciences	553311
Hamster Anti-/Mouse CD11c PE	HL3	BD biosciences	553802
Rat Anti-Mouse CD19 PE	6D5	BioLegend	115508
Rat Anti-Mouse CD21 FITC	7G6	BD biosciences	553818
Rat Anti-Mouse CD23 PECy7	B3B4	eBioscience	25-0232-82
Rat Anti-Mouse CD25 APC	PC61	BioLegend	102012
Rat Anti-Mouse CD25 PE	PC61.5	Tonbo Biosciences	50-0251-U100
Rat Anti-Mouse CD44 Pacific Blue	IM7	BioLegend	103020
Rat Anti-Mouse CD44 BV421	IM7	BD biosciences	563970
Mouse Anti-Mouse CD45.1 PerCPCy5.5	A20	Tonbo Biosciences	65-0453-U100
Mouse Anti-Mouse CD45.1 APC	A20	eBioscience	17-0453-82
Mouse Anti-Mouse CD45.1 FITC	A20	BD Pharmingen	553775
Mouse Anti-Mouse CD45.1 PE	A20	Tonbo Biosciences	50-0453-U-100
Mouse Anti-Mouse CD45.2 PECy7	104	Tonbo Biosciences	60-0454-U100
Mouse Anti-Mouse CD45.2 Pacific Blue	104	BioLegend	109820
Mouse Anti-Mouse CD45.2 FITC	104	BD Pharmingen	553772
Mouse Anti-Mouse CD45.2 BV737	104	BD biosciences	612778
Rat Anti-Mouse CD62L PE	MEL-14	eBioscience	12-0621-83
Rat Anti-Mouse CD62L BV711	MEL-14	BioLegend	104445
Rat Anti-Mouse CD69 PECy7	H1.2F3	BioLegend	104502
Rat Anti-Mouse CD73 BV605	TY/11.8	BioLegend	127215
Rat Anti-Mouse CD86 Pacific Blue	GL-1	BioLegend	105022
Rat Anti-Mouse CD93 (AA4.1)	AA4.1	eBioscience	17-5892-82
Rat Anti-Mouse CD138 PE	281-2	BioLegend	142504
Hamster Anti-Mouse CD95 (Fas) PECy7	Jo2	BD Pharmingen	557653
Rat Anti-Mouse CD185 (CXCR5) BV605	L138D7	BioLegend	145513
Rat Anti-Mouse FR4 (Folate Receptor 4) PECy7	12A5	BioLegend	125012
Hamster Anti-Mouse γδ TCR PE	GL3	BD Biosciences	553178
Rat Anti-Mouse GL7 PerCPCy5.5	GL7	BioLegend	144610
Rat Anti-Mouse Ly-6g (Gr1) PE	1A8	BD Biosciences	551461
Rat Anti-Mouse IgD APC-eFluor 780	11-26c	eBioscience	47-5993-82
Rat Anti-Mouse MHC Class II APC	M5/114.15.2	Tonbo Biosciences	20-5321-U100
Rat Anti-Mouse NK-1.1 PE	PK136	BD Biosciences	553165
Rat Anti-Mouse CD279 (PD-1) PCPCy5.5	29F.1A12	BioLegend	135208
Rat Anti-Mouse CD49b (pan NK cell marker) PE	DX5	BD Biosciences	553858

Antibodies for intra-cellular staining

Antibody	Clone	Vendor	Catalog number
Rat Anti-Mouse Foxp3 APC	FJK-16s	eBioscience	17-5773-82
Rat Anti-Mouse Foxp3 FITC	FJK-16s	eBioscience	11-5773-82
Rabbit Anti-active Caspase-3 Alexa Fluor 647	C92-605	BD Biosciences	560626
Mouse Anti-Mouse NUR77 PE	12.14	eBioscience	12-5965-80
Rat Anti-Mouse IL-2 PE	JES6-5H4	eBioscience	12-7021-41
Rabbit Phospho-p44/42 MAPK (T202/Y204) unconjugated	197G2	Cell Signaling	4377S/4377L
Goat Anti-Rabbit IgG (H+L) APC	Polyclonal	Jackson ImmunoResearch Laboratories	111-005-144

Stimulatory Antibodies

Antibody	Clone	Vendor	Catalog number
Purified anti-mouse CD3ε Antibody	2c11	BioLegend	100340
Purified anti-mouse CD28 Antibody	37.51	BioLegend	102102
AffiniPure Goat Anti-Armenian Hamster IgG (H+L)	Polyclonal	Jackson ImmunoResearch Laboratories	127-005-099

Secondary Antibody for the detection of anti-nuclear antibody

Antibody	Clone	Vendor	Catalog number
FITC Donkey Anti-Mouse IgG (H+L)	Polyclonal	Jackson ImmunoResearch Laboratories	715-095-150
